# Bipolar haemostatic forceps versus standard therapy by haemoclip + / − epinephrine injection as initial endoscopic treatment in active non-variceal upper GI bleeding: study protocol for a prospective, randomized multicentre trial (BeBop-Trial)

**DOI:** 10.1186/s13063-023-07394-x

**Published:** 2023-06-15

**Authors:** Daniel Schmitz, Lucas Thielemann, Felix Grassmann

**Affiliations:** 1 Department of Gastroenterology and Infectiology, Helios Kliniken Schwerin, University Campus of Medical School Hamburg, Wismarsche Str.393-397, Schwerin, 19055 Germany; 2 Department of Medical Statistics and Epidemiology, Medical School Hamburg, Am Kaiserkai 1, Hamburg, 20457 Germany

**Keywords:** Haemostasis, Endoscopic, Gastrointestinal haemorrhage, Randomized controlled trial

## Abstract

**Background:**

Patients with active nonvariceal upper gastrointestinal bleeding (NVUGIB) usually require urgent endoscopic treatment. Standard therapy (ST) using haemoclip + / − epinephrine injection is not always successful. Bipolar haemostatic forceps (HemoStat/Pentax®) are an approved medical device for the treatment of gastrointestinal bleeding. However, their use as a primary endoscopic treatment for active NVUGIB has not yet been proven in a randomized prospective study.

**Methods:**

This is a prospective, randomized, multicentre superiority trial (*n* ≥ 5). Patients with active NVUGIB will be randomized (1:1) to ST and to experimental therapy (ET) by application of bipolar haemostatic forceps.

In the case of failed initial treatment within 15 min, crossover treatment will be attempted first. Rescue treatment (e.g. via over-the-scope-clip) will then be allowed after 30 min. All patients will also receive standard therapy with proton pump inhibitors. Forty-five patients per treatment arm are required to demonstrate an absolute difference of 25.4% with a power of 80% and a significance level of 0.05.

**Discussion:**

The hypothesis of the study is that bipolar haemostatic forceps are superior to ST in terms of successful primary haemostasis and the absence of recurrent bleeding within 30 days (combined endpoint). The 1:1 randomization is also ethically justifiable for this study, as both procedures are approved for the intervention in question. To further increase the safety of the patients in the study, crossover treatment and rescue treatment are planned. The prospective design seems feasible in a reasonable time frame (recruitment period of 12 months), as nonvariceal upper gastrointestinal bleeding is common. Anticoagulants and/or antiplatelet drugs could be an important confounding factor in the statistical analysis that needs to be taken into account and calculated if necessary. In conclusion, this randomized, prospective, multicentre study could make an important contribution to answering the question of whether bipolar haemostatic forceps could be the first-line therapy in the endoscopic treatment of stage Forrest I a + b NVUGIB.

**Trial registration:**

ClinicalTrials.gov NCT05353062. Registered on April 30 2022.

**Supplementary Information:**

The online version contains supplementary material available at 10.1186/s13063-023-07394-x.

## Administrative information

**Table:** 

Title {1}	Bipolar haemostatic forceps versus standard therapy by a haemoclip + / − epinephrine injection as initial endoscopic treatment in active nonvariceal upper GI bleeding: A prospective, randomized multicentre study (BeBop-Trial)
Trial registration {2a and 2b}	ClinicalTrials.gov NCT05353062 (30/04/2022)
Protocol version {3}	2.3 from 19.12.2022
Funding {4}	PENTAX Medical Europe, Julius-Vosseler-Straße 104, 22527 Hamburg, Germany (only financial support)
Author details {5a}	- Dr. med. Daniel Schmitz, Department of Gastroenterology and Infectiology, Helios Kliniken Schwerin, Schwerin, Germany - Lucas Thielemann, Department of Gastroenterology and Infectiology, Helios Kliniken Schwerin, Schwerin, Germany - Prof. Dr. Felix Grassmann, Institute for Medical Statistics and Epidemiology, Medical School Hamburg, Hamburg, Germany
Name and contact information for the trial sponsor {5b}	Helios Kliniken Schwerin, Department of Gastroenterology and Infectiology, Wismarsche Str. 393 – 397, 19055 Schwerin, Germany
Role of sponsor {5c}	The study sponsor (Helios Kliniken Schwerin) and the funder (Pentax Medical Europe) did not have and will not have any authority over the study design, data collection, data management or data analysis as well as interpretation of data, writing of the report and decision to submit the report for publication

## Introduction

### Background and rationale {6a}

The most common causes of acute upper gastrointestinal bleeding are nonvariceal. These include gastric and duodenal ulcers, erosive mucosal diseases of the oesophagus, stomach or duodenum, malignant diseases, Mallory-Weiss syndrome, Dieulafoy lesions, unidentifiable causes [1], or bleeding after endoscopic interventions such as polypectomy, endoscopic mucosal resection (EMR), or endoscopic submucosal dissection (ESD) [2].

The European Society of Gastrointestinal Endoscopy (ESGE) recommends a combination of thermal or mechanical therapy + / − epinephrine injection for patients with actively bleeding ulcers (Forrest I a and I b) in the updated guidelines on nonvariceal upper gastrointestinal bleeding published in 2021 [3]. Usually, a haemoclip is used as mechanical therapy. According to the GRADE criteria (Grading of Recommendations, Assessment, Development and Evaluation [4], the level of recommendation is strong and based on high-quality scientific evidence. However, as these methods are sometimes insufficiently effective in high-risk situations, the guideline recommends the use of an over-the-scope clip (OTSC) for actively bleeding ulcers > 2 cm in size that have a large visible vessel > 2 mm or are in a high-risk vascular location (e.g. gastroduodenal or left gastric artery), or for excavated/fibrotic ulcers, although no prospective randomized studies are available [3]. The most recently published studies on thermal haemostasis have predominantly used monopolar haemostatic forceps [5–8]. However, all these studies also included ulcers that were not actively bleeding (Forrest II and/or Forrest II b), which reduces the clear definition of the efficacy of the respective haemostatic method, as not all ulcers in stage Forrest II a or b bleed again. Only in a small, nonrandomized study of 50 patients was the use of bipolar haemostatic forceps more effective than endoscopic clipping in terms of initial haemostasis. However, no statistical superiority was shown in the prevention of recurrent bleeding [9]. Nonbleeding ulcers in stage Forrest II a and tumour bleeding, which often cannot be satisfactorily treated endoscopically, were also included. The bipolar haemostatic forceps from Pentax [10] has already been approved for the treatment of bleeding in the gastrointestinal tract in Europe as well as in Germany. Moreover, it has been successfully used to treat blood vessels or active bleeding during endoscopic submucosal dissection (ESD) and third-space endoscopy, such as peroral endoscopic myotomy (POEM). In contrast to monopolar forceps, there is no need to attach an earthing cable to the patient, as the current flow is directly between the opened branches of the forceps and not through the entire body. This also prevents electrical interference with implanted pacemakers or cardioverter defibrillators. Furthermore, only superficial tissue layers are destroyed by the horizontal current flow, which might reduce the risk of perforation. In summary, there is an unmet need for an easy-to-use and safe haemostasis method that achieves a very high initial haemostasis rate, prevents rebleeding in the long term, and can be used in all sites that can be reached with conventional oesophagogastroduodenoscopy. Pentax bipolar haemostatic forceps may offer a solution to this need. Its use has not yet been demonstrated in a prospective randomized study with a statistically sufficient number of patients.

### Objectives {7}

The efficacy and safety of bipolar haemostatic forceps should be investigated in the primary treatment of active, nonvariceal upper gastrointestinal haemorrhage of any cause (except tumour haemorrhage) in a randomized, prospective, multicentre superiority trial. In this setting, bipolar haemostatic forceps are the experimental study arm, and the standard therapy of haemoclip + / − epinephrine injection is the comparator study arm. The primary objective is the combined endpoint of successful initial endoscopic haemostasis and the absence of rebleeding within 30 days after successful initial haemostasis. Secondary objectives are the number of salvage procedures such as OTSC, angiography + coiling or surgery for primary haemostasis, type and number of therapy-associated complications, number of endoscopic reinterventions in 30-day intervals, number of blood units transfused, length of hospital stay, length of intensive care unit stay, and 30-day mortality.

### Trial design {8}

The trial design is as follows: prospective, multicentre superiority trial with 1:1 randomization of standard therapy versus experimental therapy.

## Methods: participants, interventions, and outcomes

### Study setting {9}

At least five study centres in Germany are participating. The study centres consist of university hospitals as well as community hospitals. The coordinating study centre is Helios Kliniken Schwerin, the university campus of Medical School Hamburg. The actual study centre list is available at the coordinating study centre study centre on request.

### Eligibility criteria {10}

Inclusion criteria:

- Age ≥ 18 years
- Active, nonvariceal upper gastrointestinal haemorrhage in the oesophagus, stomach, or duodenum (including metachronous, postinterventional haemorrhage): Forrest I a bleeding = spurting or pulsatile bleeding or Forrest I b bleeding = oozing bleeding
- Written consent of the patient or available authorized surrogate
- Life expectancy of at least 30 days

Exclusion criteria:

- Nonactive, nonvariceal upper gastrointestinal bleeding stage Forrest II a-c or Forrest III (ulcer)
- Variceal haemorrhage = bleeding from varices in the oesophagus, stomach, or duodenum
- Tumour haemorrhage
- Severe coagulation disorder not responding to transfusion of blood products: platelet count < 50.000/μl and/or INR > 3.0 and/or PTT > 2 × the normal value (> 52–72 s depending on reagent)
- Emergency that precludes endoscopy (for example, perforation in the gastrointestinal tract)
- Pregnant or breastfeeding patient

Only gastrointestinal endoscopists with more than 5 years of experience in emergency endoscopy who are familiar with both methods of haemostasis may perform the study therapy.

### Who will take informed consent? {26a}

Informed consent from potential trial participants or authorized surrogates will be obtained by the investigator before application of the study therapy.

### Additional consent provisions for collection and use of participant data and biological specimens {26b}

N/A. Additional collection and use of participant data for other reasons as well as collection of biological specimens are not provided.

## Interventions

### Explanation for the choice of comparators {6b}

The European Society of Gastrointestinal Endoscopy (ESGE) recommends a combination of thermal or mechanical therapy + / − epinephrine injection for patients with actively bleeding ulcers (Forrest I a and I b) in the updated guidelines on nonvariceal upper gastrointestinal bleeding published in 2021 [3]. Therefore, haemoclip + / − epinephrine injection is used as a comparator.

## Intervention description {11a}

### Standard intervention

The bleeding vessel is clamped with a clip, which remains in the patient. Since clips from different manufacturers (Boston®, Cook®, MICRO TECH® and others) are largely equally effective, the use of a specific product is not intended in the study. In most cases, the clip falls off spontaneously after a few days and is later excreted with bowel movements. Injection with adrenaline solution at a dosage of 1:10,000 in 10 ml (= adrenaline 0.1 mg/ml) can produce an additive effect on haemostasis by vasoconstriction and by “cushion” vascular compression. Its use is optional.

### Experimental intervention

Bipolar haemostatic forceps are placed directly on the bleeding vessel with the forceps open, and current is applied, flowing between the open forceps arms. The current application is triggered by the examiner via a foot pedal that is connected to the generator. The current is applied with short pulses of a few seconds until haemostasis is complete.

**Fig. 1:**
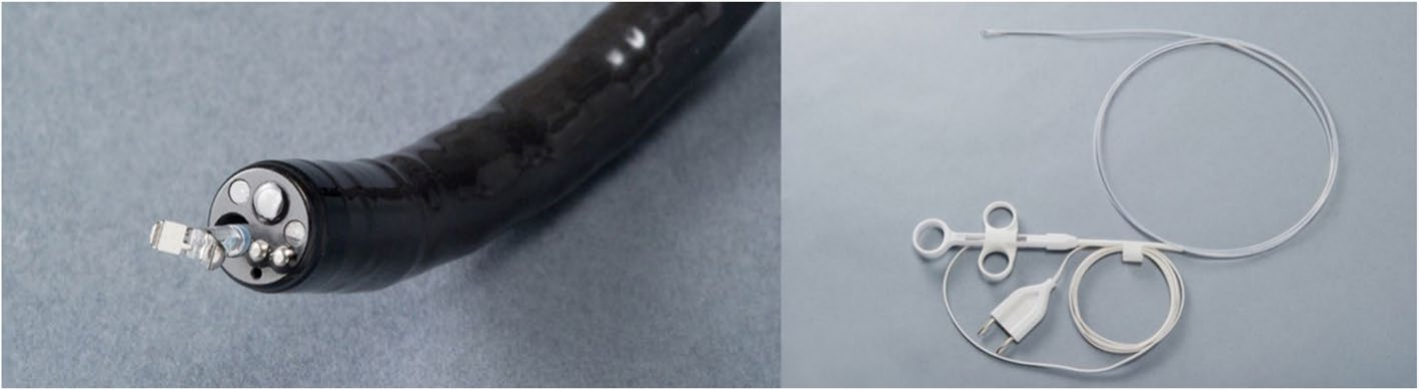
Pentax HemoStat-WideCup bipolar haemostatic forceps inserted in the endoscope (left) and shown as a whole catheter (right)

The HemoStat-WideCup bipolar haemostatic forceps from Pentax Medical (Fig. 1) [10] is a therapeutic medical device already certified for use in the gastrointestinal tract.

**Fig. 2:**
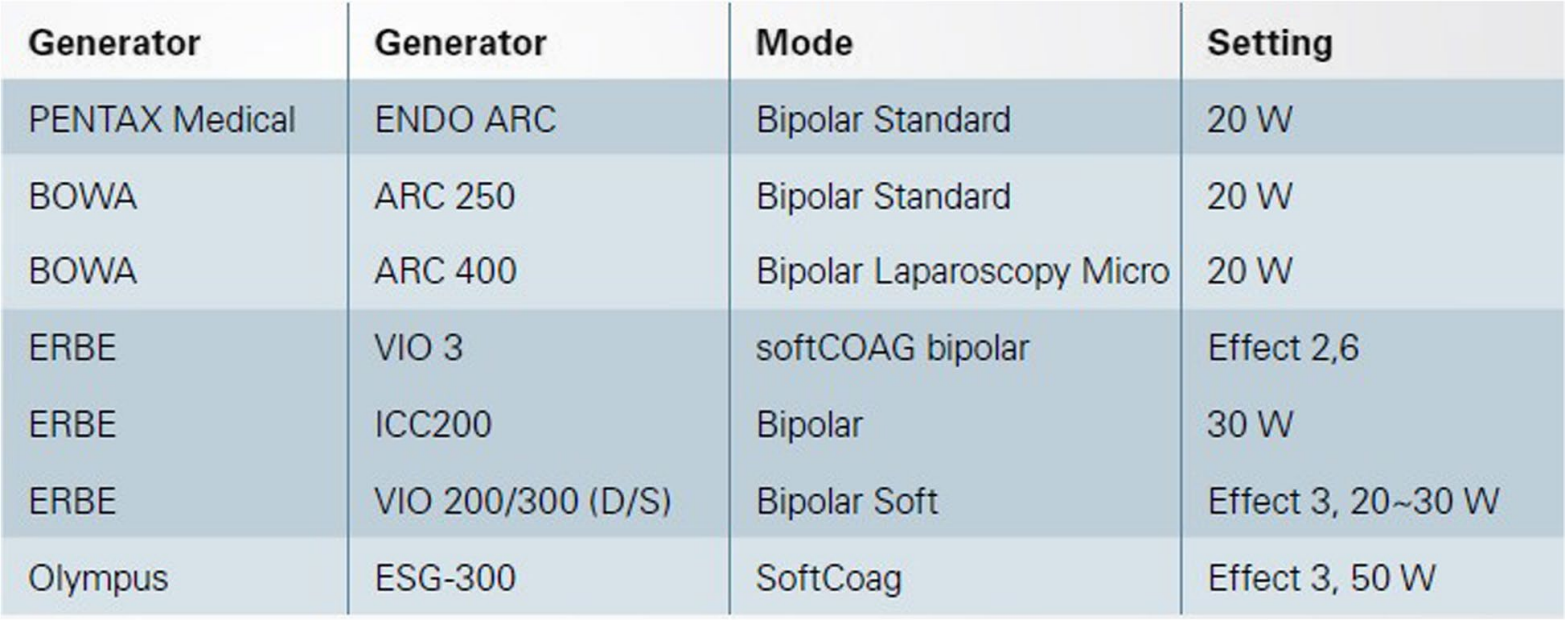
Settings of the HemoStat WideCup bipolar electric haemostatic forceps for different generators

The settings on the connected current generator may differ. The manufacturer recommends the following settings, which can also be saved as a separate setting programme in the generator if necessary (Fig. 2). The settings may be modified by the user. Bipolar electric forceps must not be used in the direct vicinity of flammable gases, liquids, or substances with a risk of fire or explosion, which does not occur in the body during regular use.

Both experimental therapy (ET) and standard therapy (ST) are used primarily with the aim of stopping bleeding within a period of 15 min after locating the source of bleeding.

### Criteria for discontinuing or modifying allocated interventions {11b}

If no haemostasis is achieved, it should be attempted in the following 15 min with the opposite method (ET after ST or ST after ET) (= crossover treatment). If no haemostasis is achieved after a total time of approximately 30 min, alternative haemostasis methods such as topical haemostatic spray/powder, over-the-scope clip, angiographic coiling, or surgery can be used (= rescue treatment).

### Strategies to improve adherence to interventions {11c}

The investigator will check daily during the hospital stay as to whether coagulation medication has been started and discontinued according to the guidelines. He will also check daily to determine whether the proton pump inhibitors prescribed in the study protocol have been taken. At the follow-up examination after 30 days, the patient will be asked whether the prescribed proton pump inhibitors have been taken. Moreover, the patient will be informed that taking proton pump inhibitors is important to fulfil the criteria of the study protocol.

### Relevant concomitant care permitted or prohibited during the trial {11d}

All patients with suspected nonvariceal upper gastrointestinal bleeding will receive an intravenous bolus of the proton pump inhibitor pantoprazole 80 mg prior to the endoscopic examination according to the guidelines, followed by an intravenous bolus or short infusion of 40 mg/day 3 times a day or continuous intravenous administration with a dosage of 240 mg pantoprazole/day for 3 days. In the case of ulcer bleeding, the proton pump inhibitor pantoprazole 40 mg orally in tablet form will be continued at a dosage of 1-0-1/day for the entire follow-up period of 30 days (or beyond, depending on the requirements of the treating physician).

Discontinuation and resumption of anticoagulant medication will be conducted according to the guidelines of the European Society of Gastrointestinal Endoscopy (ESGE) [12]. As a rule, medication with an anticoagulant drug should be resumed no later than 48 h after successful haemostasis if the indication persists. The procedure for exceptions to this rule, for example, in the presence of mechanical heart valves, can be found in the guideline. The anticoagulant medication will be carefully documented over the entire examination period from the initial emergency endoscopy to the end of the follow-up period after 30 days.

### Provisions for post-trial care {30}

N/A as ancillary and post-trial care are not planned, and additional assurance for trial participation is not necessary, as both therapies are approved.

### Outcomes {12}

All efficacy and safety parameters will be documented in a case report form (CRF) developed for the study.

#### Primary outcome measure

1. Success of primary haemostasis
  Period: 15 min from localization of the source of bleeding until haemostasis
  Method of measurement: assessment by endoscopist: yes/no
  Explanation: variable of immediate effectiveness
2. Number of recurrent haemorrhages within 30 days
  Period: 30 days after initial haemostasis
  Method of measurement: evidence of recurrent gastrointestinal bleeding on oesophagogastroduodenoscopy or clinical signs of upper gastrointestinal bleeding (such as tarry stools) and an associated drop in haemoglobin of > 1 g/dl or 0.62 mmol/l
  Explanation: variable of sustained efficacy in an appropriate observation period, as recurrent bleeding is not uncommon after endoscopic haemostasis

#### Secondary and other outcomes

1. Type and number of therapy-associated adverse events
  Period: 30 days
  Measurement method: description and count
  Explanation: parameters of safety of haemostasis methods
2. Number and success of crossover treatment
  Period: 30 min after localization of the source of bleeding
  Measurement method: counting and assessment by the endoscopist: yes/no
  Explanation: further method to assess the efficacy of the haemostasis intervention with due regard to patient safety
3. Type, number, and success of rescue treatment
  Period: up to 6 h after localization of the source of bleeding
  Method of measurement: assessment, counting and evaluation by the investigator: yes/no
  Explanation: Further method to assess the efficacy of the haemostasis intervention with due regard to patient safety
4. Number of endoscopic reinterventions
  Period: 30 days after initial successful endoscopic haemostasis
  Measurement method: count of further endoscopic haemostasis procedures performed
  Explanation: indirect test of the effectiveness of the primary haemostasis method
5. Number of blood units transfused
  Period: 30 days after successful initial haemostasis
  Method of measurement: count of infused blood units (transfusion unit) per patient
  Explanation: indirect test of the effectiveness of the primary haemostasis method
6. Length of hospital stay
  Period: 30 days after successful initial haemostasis
  Measurement method: count of hospital days rounded up to the nearest whole day
  Rationale: indirect assessment of the effectiveness of the primary haemostasis method
7. Length of the intensive care unit (ICU) stay
  Period: 30 days after successful initial haemostasis
  Method of measurement: count of the ICU days rounded up to the nearest whole day
  Explanation: indirect test of the effectiveness of the primary haemostasis method
8. 30-day mortality
  Period: 30 days after successful initial haemostasis
  Method of measurement: count of patients who die during the interval, regardless of the cause of death
  Explanation: indirect test of the effectiveness of the primary haemostasis method
9. Time until reaching complete initial haemostasis
  Period: 15 min after starting initial haemostasis
  Method of measurement: time measurement in minutes
  Explanation: indirect test of the effectiveness of the primary haemostasis method

### Participant timeline {13}

The time schedule of enrolment, interventions, and assessments are shown in a schematic diagram (Table 1).

**Table 1:**
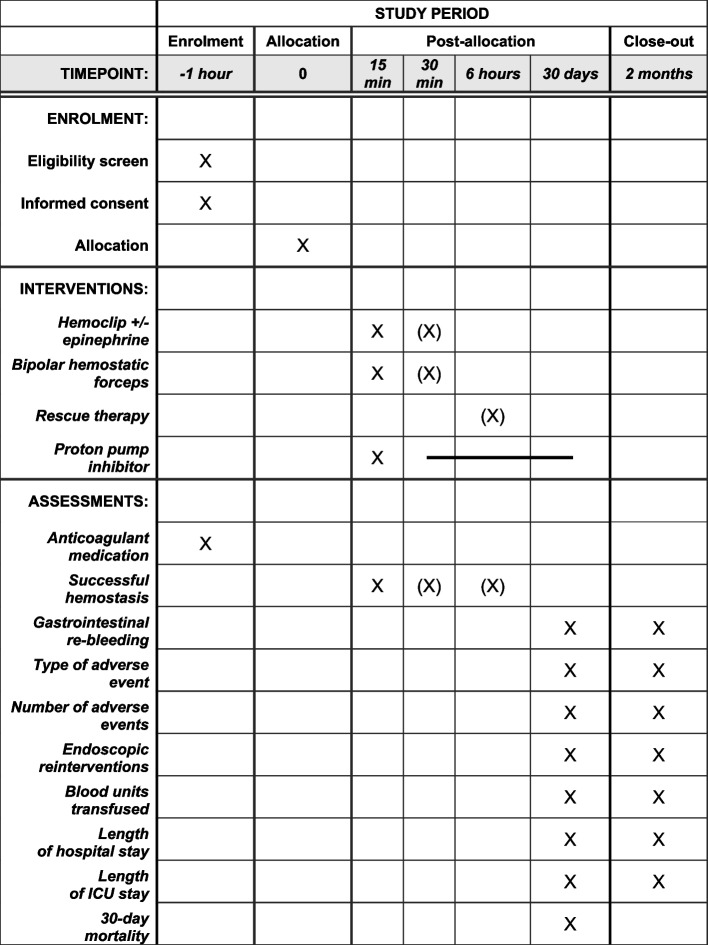
Participant timeline. Haemoclip + / − epinephrine injection and bipolar haemostatic forceps are only used as crossover treatment if successful haemostasis has not been achieved after 15 min (X). Rescue therapy (OTSC, coiling, surgery) is only applied if successful haemostasis has not been achieved after a further 15 min by crossover treatment (X)

### Sample size {14}

The study hypothesis is that experimental therapy is superior to standard therapy in terms of clinical success, defined as successful haemostasis and no (signs of) rebleeding within 30 days. In one study, the rates of successful haemostasis and rebleeding in the experimental group were 92.3% and 0%, respectively [9]. However, this nonrandomized trial also included nonactive nonvariceal bleeding. Therefore, the rate of successful haemostasis could be lower and the rate of rebleeding higher when only active nonvariceal bleeding is present. Therefore, a success rate of 90% for primary haemostasis and a rebleeding rate of 5% were assumed, giving a combined clinical success rate of 85.5%. In a recently published study comparing the Over-the-Scope Clip (OTSC) with the haemoclip in high-risk patients with acute nonvariceal upper gastrointestinal bleeding, the rates of successful haemostasis and rebleeding in the standard of care arm (haemoclip only) were 73.1% and 15.4%, respectively [12]. However, this randomized trial also included nonactive, nonvariceal bleeding. Therefore, the rate of successful haemostasis could be lower and the rate of rebleeding higher if only active nonvariceal bleeding is present. Therefore, a success rate of 72% for primary haemostasis and a rebleeding rate of 16% were assumed, resulting in a combined clinical success rate of 60.1%. Power analysis of the combined primary endpoint revealed that 45 patients per treatment arm are required to demonstrate an absolute difference of 25.4 percentage points (85.5–60.1%) with a power of 80% and a significance level of 0.05. Here, a simulation-based line calculation assuming the above absolute differences was performed using the SimEngine package version 1.1.0, implemented in R version 3.3.0. Overall, a dropout rate of a maximum of 10% is assumed, as the follow-up period is only 30 days. A decision on the recruitment of follow-up patients will be made when 90 patients have been included as planned and the follow-up observations are available.

### Recruitment {15}

Active upper gastrointestinal bleeding without varices is a frequent indication for emergency endoscopy and occurs approximately once a week in the coordinating study centre (Helios Kliniken Schwerin, Germany). It is therefore expected that 90 patients in five study centres will be reached over a period of 12 months. An interim analysis of the recruited patient number will be conducted after 6 months. If necessary, further study centres must be included to achieve enough participants to reach the target sample size.

## Assignment of interventions: allocation

### Sequence generation {16a}, concealment mechanism {16b}, and implementation {16c}

For randomization, computer-generated random number sequences are generated at the study centre in Schwerin, with odd numbers representing experimental therapy (ET), bipolar electrical haemostasis forceps (HemoStat/Pentax), and even numbers representing standard therapy (ST). Randomization will be performed in blocks of 4 and stratified per centre. Trained study nurses will prepare sealed envelopes and distribute them to the participating centres. Patients meeting the inclusion criteria will be randomized 1:1 during endoscopy, as ascertained by opening the sealed envelopes. Randomization will be performed by the treating endoscopist. The following rules will be applied: the person who prepares the envelopes and conducts the allocation is not involved in the study. The letters are numbered consecutively and are not transparent. The person who opens the letter is not the same person who has prepared the envelopes (endoscopy assistant) [13].

## Assignment of interventions: blinding

### Who will be blinded {17a}

The study participants will be blinded after being assigned to the interventions. The endoscopic haemostatic method is performed under short sedation (usually propofol sedation). Therefore, the assignment to the intervention cannot be observed by the study participants. Furthermore, the participants are not informed on the assigned treatment arm until the end of the follow up-period of 30 days. The endoscopist cannot be blinded, as he must be aware of which endoscopic intervention he is performing on the patient. Physicians on the ward are not blinded as the endoscopic report will comprise the assigned intervention. On the other hand, outcome assessors will be blinded.

### Procedure for unblinding if needed {17b}

In the case of a life-threatening emergency, both the patient and subsequent clinicians are unblinded if information about the treatment group is necessary (e.g. the fact whether clips are present or not).

## Data collection and management

### Plans for assessment and collection of outcomes {18a}

All data collected as part of the study will be documented on a standardized paper-based case report form (CRF) version 2.2 (03/12/2022) (Additional file 1). The investigator is responsible for ensuring that all parts of the CRFs are completed correctly. Errors made in completing the CRFs should be crossed out single-spaced in accordance with ICH-GCP [14] so that the original entry remains legible; the new entry should be dated and initialled with the investigator’s name. In the case of self-explanatory corrections (e.g. numerical error in the date), the justification may be omitted. Each CRF must be signed at least once by the investigator. The completed pages of the CRF will be sent to the lead study centre. A copy will remain at the study centre.

### Plans to promote participant retention and complete follow-up {18b}

Study participants will be informed and must provide written consent to be contacted by telephone or in writing by the investigator after 30 days to complete follow-up data. A separate list will be provided for participants who cannot be contacted after 30 days or who have deviated from the intervention protocols.

### Data management {19}

Database set-up, double-entry data collection, data storage, and data validation are the responsibility of the principal investigator’s study centre. All data management processes will be performed according to standard operating procedures (SOPs). Data validation includes checking the completeness, consistency and plausibility of the data documented in the CRF. For this purpose, a query system will be set up between data management and the investigator. In the so-called query process, requests for clarification of incomplete, implausible, and/or inconsistent data will be made as quickly as possible to the investigator by the data management team. These queries will be answered by the investigator or a person appointed by the investigator and transmitted to the data management team for entry into the database. After all queries for all included patients have been clarified, the database will be closed at the end of the study and handed over to the biometrician for evaluation. After completion of all evaluations and preparation of the final report, the originals of all CRFs will be transferred to the PI for archiving. After completion of the study, the data will be converted into different data formats (e.g. csv files) to ensure further use. The primary data on which the scientific publications are based will be publicly accessible for reanalyses and meta-analyses in the BioStudies public repository [15] after completion of the study.

### Confidentiality {27}

During the study, medical findings and personal information of the patient will be collected and stored in writing or electronically at the study site. The data important for the study will be additionally stored and evaluated in pseudonymised form. Pseudonymized means that no names or initials are used, but only a numerical and/or letter code, possibly with an indication of the year of birth. The data will be secured against unauthorized access. The patient has the right to information about the data stored about him or her (Art. 15 DS-GVO). If it is established that incorrect personal data regarding a patient are being processed, he or she may request that it be corrected (Art. 16 DS-GVO). He or she also has the right to request the deletion of his or her personal data if certain reasons for deletion exist. This is the case, for example, if the personal data are no longer necessary for the purpose for which they were originally collected or processed or if he or she withdraws his or her consent and there is no other legal basis for the processing (Art. 17 DS-GVO). The patient also has the right to restrict the processing of his or her personal data (Art. 18 DS-GVO), a right to data portability (Art. 20 DS-GVO), and a general right of objection (Art. 21 DS-GVO). For enquiries and complaints in this regard, the patient will be given the relevant contact addresses along with the other study information. This includes the contact data of the hospital, the investigators, the data protection officer, and the State Commissioner for Data Protection of the study centre.

### Plans for collection, laboratory evaluation, and storage of biological specimens for genetic or molecular analysis in this trial/future use {33}

N/A, as no biological samples will be collected from the study participants.

## Statistical methods

### Statistical methods for primary and secondary outcomes {20a} and for additional analyses (e.g. subgroup analyses) {20b}

An intention-to-treat analysis will be performed. Continuous variables will be reported as medians with their ranges (minimum and maximum), while categorical variables will be reported as frequencies and percentages, unless otherwise stated. For continuous variables, differences between treatment arms will be analysed using linear regression, potentially correcting for confounders such as study centre, age, anticoagulation and/or antiplatelet drugs, Forrest classification, ulcer size and location, *Helicobacter pylori* positivity, prior endoscopic treatment, and standard or bipolar haemostatic forceps therapy; categorical and binary outcomes will be analysed using logistic regression, also potentially correcting for the above confounders. Bonferroni correction will be applied to subanalyses of statistically significant tests. Corrected *p* values < 0.05 will be considered significant. Variables with a *p* value < 0.05 in the univariate model will be entered into a multivariate stepwise logistic regression model (forwards selection, likelihood ratio) to assess the independent predictive effect of the variables of interest. Statistical analysis will be performed using SPSS (version 24.0; IBM, New York, NY), Stata software (V.15; StataCorp, College Station, TX), or R (version 3.3.0 or newer).

### Interim analyses {21b}

A formal interim analysis is not planned. Since the ET is already an approved medical device for the indication stated in the study, it is not expected that serious safety-relevant events will be observed. Therefore, no data safety and monitoring committee will be established. The steering committee will decide on the extension of the planned recruitment period based on the recruitment figures.

### Methods in analysis to handle protocol nonadherence and any statistical methods to handle missing data {20c}

N/A, as protocol nonadherence and missing data are not expected as the intervention is performed by the endoscopist and the follow-up period is short (30 days).

### Plans to give access to the full protocol, participant level-data and statistical code {31c}

The full study protocol is planned to be published in an international PubMed-registered journal (e.g. Trials). The anonymized participant-level data will be publicly accessible in the BioStudies public repository [15] after completion of the study.

## Oversight and monitoring

### Composition of the coordinating centre and trial steering committee {5d}

A steering committee (SC) will be set up, which will monitor the study together with the PI and decide on all issues or on issues to be defined, e.g. by majority vote. The composition of the steering committee is as follows: Chairperson Prof. Dr. Jörg-Peter Ritz (Visceral surgeon, Helios Kliniken Schwerin), Dr. med. Gaston Schley (Dermatologist, Helios Kliniken Schwerin), and Herr Dr. med. Jochen Facklam (Thoracic surgeon, Helios Kliniken Schwerin). Other responsibilities within the scope of the study are listed below:

- Project management: Dr. med. Daniel Schmitz (PI), Helios Kliniken Schwerin
- Data management: Lucas Thielemann (doctoral candidate), Helios Kliniken Schwerin
- SAE management: Prof. Dr. Jörg-Peter Ritz, Helios Kliniken Schwerin
- Scientific advice: Prof. Dr. Christian Prinz, Helios Kliniken Wuppertal

### Composition of the data monitoring committee, its role and reporting structure {21a}

N/A. A data monitoring committee is not planned, as there will be only one follow-up interview after 30 days asking about rebleeding.

### Adverse event reporting and harms {22}

Adverse events (AEs) will be documented at the start of oral endoscope insertion and will end 30 days after the first haemostatic procedure. The type and number of adverse events are secondary outcome variables as defined above. Subsequent adverse events may no longer be directly related to details of the study or procedure. Overall, adverse events attributable to ST or ET are expected to be rare [3, 9]. An adverse event is defined as a serious adverse event (SAE) if it is fatal, life-threatening, or results in permanent or severe disability or incapacity. If an adverse event is classified as serious, it will be documented on a separate SAE form in addition to the AE documentation in the CRF. Patients will be asked at the 30-day query if any adverse or serious adverse events occurred. Adverse events will be documented in the CRF with the following parameters: date and time of occurrence and cessation, association with trial therapy, and outcome of the adverse event. Conditions that existed prior to the administration of the investigational product will be documented not as adverse events but as concomitant conditions. Any new disease or any disease that increases in severity during the trial shall be documented as an adverse event. Each adverse event shall be assessed by the investigator as to whether a relationship to the intervention can be suspected. The nature and pattern of the reaction, the temporal relationship with the intervention, the clinical condition of the patient, concomitant medication, and other relevant clinical parameters should be considered. If the event occurs due to lack of efficacy or due to the underlying disease, it shall be considered independent.

The following definitions are used to assess the causality of the adverse event with the intervention (WHO Causality Assessment of Suspected Adverse Reactions) [16]:

- Certain: an event directly related to the intervention and observed, e.g. organ perforation
- Probable: an event that has a traceable temporal relationship to the use of the test therapy, e.g. a temporally displaced organ perforation
- Possible: an event that is reasonably temporally related to the use of the intervention but could easily have been caused by a number of other factors, e.g. a heart attack
- Unlikely: an event for which there is sufficient information to suggest that it is not related to the intervention, such as skin exanthema
- Not assessed: an event reported as an adverse event for which no assessment of association was made at the time of reporting because further data are needed or are currently being collected
- Not assessable: an assessment of the association is not possible

Regardless of the presumed causal relationship, each SAE must be documented and reported to the coordinating body. The PI, the steering committee, and the person responsible for SAE management jointly decide whether a report must be made to the medical device manufacturer and to the Federal Institute for Drugs and Medical Devices (BfArM).

### Frequency and plans for auditing trial conduct {23}

N/A, as both interventions are approved procedures or medical devices.

### Plans for communicating important protocol amendments to relevant parties (e.g. trial participants, ethical committees) {25}

Important protocol modifications will be communicated to the relevant parties of the multicentre study (e.g. investigators and trial participants), and an amendment will be requested from the relevant ethical committee.

### Dissemination plans {31a}

Within 1 year after completion of the clinical trial, the summary of the final report on the clinical trial, which includes all significant events of the study, will be submitted to the responsible ethics committee. It is planned to present the results of the clinical trial in a PubMed-listed scientific journal and/or at German and international congresses after consultation with the sponsor (Helios Kliniken Schwerin), the PI, and all investigators of the study centres. In principle, publication of the entire clinical trial is preferred. The “Uniform Requirements for Manuscripts Submitted to Biomedical Journals [17]” will be considered. There is no restriction on the publication of the study results.

## Discussion

The hypothesis of the study is that bipolar haemostatic forceps are superior to standard therapy with haemoclip + / − epinephrine injection in terms of successful primary haemostasis and the absence of recurrent bleeding within 30 days (combined endpoint). In a nonrandomized Japanese trial, the rate of successful primary haemostasis was 92.3%, and the rate of recurrent bleeding was 0% for bipolar haemostasis forceps [9]. However, active, nonvariceal bleeding was not considered in this study. Accordingly, successful primary haemostasis is expected to be somewhat lower and recurrent bleeding rates somewhat higher, as the method was used exclusively for active, nonvariceal bleeding in the BeBop study. In contrast, the rates of successful haemostasis and recurrent bleeding with standard therapy were 73.1% and 15.4%, respectively, in a recently published study in which the over-the-scope clip (OTSC) was the experimental arm [3]. This study also included nonactive, nonvariceal bleeding, so successful primary haemostasis may be lower and repeat bleeding rates higher when treating active, nonvariceal bleeding. The expected difference in success rate between the two procedures with respect to the combined endpoint was therefore calculated to be 25.4%, which is the basis for the case number calculation (*n* = 90). The 1:1 randomization is also ethically justifiable for this study, as both procedures are approved for the intervention in question. To further increase the safety of the patients in the study, crossover treatment and rescue treatment are planned. The prospective design seems feasible in a reasonable time frame (recruitment period of 12 months), as nonvariceal upper gastrointestinal bleeding is common [1]. Therapy with anticoagulants, antiplatelet agents, or a combination of the two is often associated with gastrointestinal bleeding [18, 19]. Therefore, anticoagulants and/or antiplatelet drugs could be an important confounding factor in the statistical analysis that needs to be considered and calculated if necessary [20]. Tumour haemorrhage is usually accompanied by diffuse bleeding at several sites and haemostasis by clip or haemostatic forceps did not appear to be promising. Therefore, tumour haemorrhage was not included in the study as it could bias the statistical results. In consequence, this study cannot make a reliable statement about the use of haemostasis forceps in terms of haemostasis of tumour bleeding in the upper gastrointestinal tract. In conclusion, this randomized, prospective, multicentre study could make an important contribution to answering the question of whether bipolar haemostatic forceps could be the first-line therapy in the endoscopic treatment of upper gastrointestinal nonvariceal bleeding stage Forrest I a + b.

## Trial status

Study protocol version 2.3 from 19 December 2022 is the currently valid version. The following is the study timeline:

- Duration of clinical phase: [12 months]
- FSI (first subject in): [01 January 2023]
- LSI (last subject in): [31 December 2023]
- LSO (last subject out): [31 January 2024]
- DBL (database lock): [31 March 2024]
- Completion of the statistical analysis: [31 May 2024]
- Completion of the study report: [31 July 2024]

### Supplementary Information

**Additional file 1.** Case Report Form (German) version 2.3 from 05 April 2023.

**Additional file 2.** Contract funder/coordinating study centre (German) from 31 December 2022.

**Additional file 3.** Contract funder/coordinating study centre (translated in English) from 31 December 2022.

**Additional file 4.** Ethical vote (German) on 19 October 2022.

**Additional file 5.** Ethical vote (translated in English) on 19 October 2022.

**Additional file 6.** Ethical vote amendment (German) on 16 January 2023.

**Additional file 7.** Ethical vote amendment (translated in English) on 16 January 2023.

## Data Availability

All study data are made available on the BioStudies public repository [15]. There are no contractual agreements restricting the investigators’ access to the study data.

## Abbreviations

BfArM: Bundesinstitut für Arzneimittel und Medizinprodukte
CRF: Case report form
DS-GVO: Datenschutz-Grundverordnung (= German Data Protection Regulation)
EMR: Endoscopic mucosal resection
ESD: Endoscopic submucosal resection
ESGE: European Society of Gastrointestinal Endoscopy
DBL: Database lock
ET: Experimental therapy
FSI: First subject in
GRADE: Grading of recommendations, assessment, development, and evaluation
ICH-GCP: International Conference on Harmonisation-Good Clinical Practice
ICU: Intensive care unit
LSI: Last subject in
LSO: Last subject out
OTSC: Over-the-scope clip
PI: Principal investigator
POEM: Peroral endoscopic myotomy
NVUGIB: Nonvariceal upper gastrointestinal bleeding
SAE: Severe adverse event
SC: Steering committee
SOP: Standard operation procedure
ST: Standard therapy
