# Correction: Bipolar haemostatic forceps versus standard therapy by haemoclip + / − epinephrine injection as initial endoscopic treatment in active non-variceal upper GI bleeding: study protocol for a prospective, randomized multicentre trial (BeBop-Trial)

**DOI:** 10.1186/s13063-023-07751-w

**Published:** 2023-11-10

**Authors:** Daniel Schmitz, Lucas Thielemann, Felix Grassmann

**Affiliations:** 1 Department of Gastroenterology and Infectiology, Helios Kliniken Schwerin, University Campus of Medical School Hamburg, Wismarsche Str.393-397, Schwerin, 19055 Germany; 2 Department of Medical Statistics and Epidemiology, Medical School Hamburg, Am Kaiserkai 1, Hamburg, 20457 Germany

**Correction: Trials 24, 407 (2023)**

**https://doi.org/10.1186/s13063-023-07394-x**

Following publication of the original article [1], we have been notified that the financial supporter of the study has changed. Therefore, Additional files 2 and 3 have been updated, as well as the Funding section.

Originally published Funding section:

Open Access funding enabled and organized by Projekt DEAL. Financial support for the study is provided by PENTAX Medical Europe, Julius-VosselerStraße 104, 22527 Hamburg, Germany. Specifcally, an expense allowance of 75 € will be paid for each patient recruited. In addition, the costs for applying for the ethical vote will be covered by Pentax. No further funding will be provided. The funder has had and will have no infuence on the study design, data collection, data management, data analysis, interpretation of the data, writing of the report, and the decision to submit the report for publication (Additional fles 2 and 3).

Corrected Funding section:

Open Access funding enabled and organized by Projekt DEAL. Financial support for the study is provided by Helios Kliniken GmbH, Germany (Grant no. 2022-0035). Specifically, an expense allowance of 75 € will be paid for each patient recruited. In addition, the costs for applying for the ethical vote will be covered by Helios Kliniken GmbH, Germany. No further funding will be provided. The funder has, had, and will have no infuence on the study design, data collection, data management, data analysis, interpretation of the data, writing of the report, and the decision to submit the report for publication (Additional files 2 and 3).

The original article has been corrected.

### Supplementary Information

**Additional file 2. **Contract funder/coordinating study centre (German) from 31 December 2022.

**Additional file 3. **Contract funder/coordinating study centre (translated in English) from 31 December 2022.
