# Supplementary material for: Bipolar haemostatic forceps versus standard therapy by haemoclip + / − epinephrine injection as initial endoscopic treatment in active non-variceal upper GI bleeding: study protocol for a prospective, randomized multicentre trial (BeBop-Trial)
Source: Trials. 2023 Jun 15;24:407. doi: 10.1186/s13063-023-07394-x (PMC10268387; doi:10.1186/s13063-023-07394-x)
Supplement: Supplementary file 1 — Additional file 1. Case Report Form (German) version 2.3 from 05 April 2023. [file 13063_2023_7394_MOESM1_ESM.docx]

**Case Report Form**

**BeBop-Studie**

Bipolare elektrische Blutstillungszange (HemoStat/Pentax) versus Standardtherapie (Clip +/- Unterspritzung mit Adrenalinlösung)

bei der endoskopischen Erstbehandlung der aktiven nicht-varikösen Blutung im oberen Verdauungstrakt

**Bitte sende die Seiten 1-4 umgehend nach Studieneinschluss per Fax/E-Mail an:**

**Fax +49-385-520-3726 oder Lucas.Thielemann@helios-gesundheit.de**

| **Patientenidentifikation** |
| --- |
| Zent Zentrumsnummer □□ Laufende Patientennummer □□ Geburtsjahr □□□□ |
| **Kontrolle der Patienteneignung/Einverständniserklärung** |
| Prüfung der Ein-/Ausschlusskriterien erfolgt ja□ nein□ |
| Schriftliche Einverständniserklärung zur Studie liegt vor ja□ nein□ |

| **Studieneinschluss/Intervention** |
| --- |
| Datum Studieneinschluss □□.□□.□□□□ |
| Durch Randomisierung zugewiesene Intervention:  Bipolare Zange □ Standardtherapie (Clip und/oder Unterspritzung) □ |

| **Bestätigung der Angaben** |
| --- |
| Name des Prüfarztes in Blockschrift |
| Datum □□.□□.□□□□ Unterschrift: |

**BeBop-Studie Ein- und Ausschlusskriterien (Visite 0)**

**Wichtig:**

Der Screeningbogen wird erst nach der Ösophagogastroduodenoskopie ausgefüllt, da sich erst bei der der endoskopischen Untersuchung entscheidet, ob eine aktive, nicht variköse Blutung vorliegt

Die folgenden Kriterien müssen alle mit „**Ja“** beantwortet werden, um in die Studie aufgenommen zu werden

| **Einschlusskriterien** | **Ja** | **Nein** |
| --- | --- | --- |
| Patient ist im Alter von ≥ 18 Jahren und zeigte in der Endoskopie eine aktive, nicht-variköse, obere gastrointestinale Blutung im Bereich des Ösophagus, Magen oder Duodenums | □ | □ |
| Forrest Ia-Blutung = sprudelnde oder pulsatile Blutung oder:  Forrest Ib-Blutung = sickernde Blutung | □ | □ |
| Eine schriftliche Einwilligung des Patienten oder der Betreuungsperson liegt vor | □ | □ |

Die folgenden Kriterien müssen alle mit **„Nein“** beantwortet werden, um in die Studie aufgenommen zu werden

| **Ausschlusskriterien** | **Ja** | **Nein** |
| --- | --- | --- |
| Variköse obere gastrointestinale Blutung im Ösophagus, Magen oder Duodenum | □ | □ |
| Nicht (mehr) aktive Blutung im Stadium Forrest II a-c (Gefäßstumpf, Blutkoagelauflagerung, Hämatinauflagerung) oder Forrest III (Ulcus) | □ | □ |
| Geschätzte Lebenserwartung von < 30 Tagen aufgrund von Multimorbidität oder fortgeschrittener Tumorerkrankung | □ | □ |
| Es lag eine Tumorblutung vor (Karzinom oder neoplastischer Polyp) | □ | □ |
| Die Patientin ist schwanger oder befindet sich in der Stillzeit | □ | □ |
| Der Patient nimmt an einer anderen interventionellen Studie zur Behandlung der oberen gastrointestinalen Blutung teil | □ | □ |

**BeBop-Studie Baseline-Patientendaten (Visite 1)**

**Demographische Daten/Anamnese**

| Geschlecht | männlich O weiblich O divers O |
| --- | --- |
| Geburtsmonat und –jahr | □□ Monat □□□□ Jahr |
| Größe | □□□cm |
| Gewicht | □□□kg KG |

**Einnahme gerinnungshemmender Substanzen in den letzten 48 Stunden vor Durchführung der Endoskopie:**

| O Aspirin  O Clopidogrel  O Prasugrel  O Ticagrelor  O Orale Antikoagulantien (OAK)  O Neue orale Antikoagulantien (NOAK) | O NSAR  O Andere gerinnungshemmende Substanzen  (bitte spezifizieren):_______________________________  O Unbekannt |
| --- | --- |

**Blutbild und Gerinnung vor Durchführung der endoskopischen Untersuchung**

| Hämoglobinwert: __________mmol/l  __________g/dl  Thrombozytenanzahl: __________/µl | PTT: _______ Sekunden  Quick: ________ %  INR: ________ |
| --- | --- |

**Wesentliche Ursache der nicht-varikösen, aktiven, oberen, gastrointestinalen Blutung**

**(keine Mehrfachnennung):**

| O Ulcus ventriculi  O Ulcus duodeni  O Mallory-Weiß-Läsion  O Erosion im Magen oder Duodenum  O Refluxösophagitis  O Cameron-Läsion im Magen | O Angiodysplasie  O Z. n. endoskopischer Intervention  (bitte spezifizieren):_______________________________  O Andere Ursache  (bitte spezifizieren):_______________________________ |
| --- | --- |

**BeBop-Studie Endoskopische Intervention (Visite 1)**

Durchgeführte endoskopische Blutstillung:

| O Bipolare Zange O Standardverfahren (Clip +/- Unterspritzung) |
| --- |

| Datum der Intervention | □□.□□.□□□□ (tt.mm.jjjj) |
| --- | --- |
| Blutstillung primär erfolgreich ? | Ja □ Nein□ |
| Wenn, nein:  Crossover-Blutstillung erfolgreich ?  (d.h. Standard nach Bipolarer Zange, bzw. Bipolare Zange nach Standard) | Ja □ Nein□ |
| Musste ein anderes Blutstillungsverfahren als Rescue-Verfahren eingesetzt werden? | Ja □ Nein□ |
| Wenn ja, welches Rescue-Verfahren (z.B. OTSC, angiographisches Coiling, Operation) ? |  |
| Rescue-Verfahren erfolgreich ? | Ja □ Nein□ |
| Trat eine Komplikation bei der endoskopischen Blutstillung auf ? | Ja □ Nein□ |
| Wenn ja, welche Komplikation (z.B. Perforation) ? |  |
| Kurze Beschreibung des Ausganges der Komplikation |  |
| Perinterventionell 80 mg Pantoprazol i.v. als Bolus erhalten ? | Ja □ Nein□ |
| Postinterventionell Pantoprazol i.v. über 72 Std.  (=3 Tage) erhalten ?  3 x 40 mg als Bolus oder 240 mg/24 Std. über Perfusor (gemäß DGVS-Leitlinie) | Ja □ Nein□ |
| Mussten perinterventionell gerinnnungsstabilisierende Substanzen gegeben werden zur definitiven Blutstillung ? | Ja □ Nein□ |
| Wenn ja, welche ? |  |

**BeBop-Studie Dokumentation des 30 Tage-Intervalls (Visite 2)**

Zentrumsnummer □□ Laufende Patientennummer □□ Geburtsjahr □□□□

| Erfolgte eine erneute endoskopische Blutstillung an der gleichen Blutungsstelle? | Ja □ Nein□ |
| --- | --- |
| Wurde eine erneute obere gastrointestinale Blutung  klinisch **+** Hämoglobinabfall > 1 g/dl oder 0,62 mmol/l diagnostiziert ? | Ja □ Nein□ |
| Erfolgte ein zeitgerechter, Wiederbeginn der klinisch indizierten medikamentösen Gerinnungshemmung (zumeist nach 48 Stunden) ? | Ja □ Nein□ |
| Wie viele Transfusionseinheiten von Erythrozytenkonzentraten wurden verabreicht nach initialer Intervention ?  (EK-Gabe muss im Zusammenhang der GI-Blutung stehen) |  |
| Helicobacter pylori nachgewiesen | Ja □ Nein□ Nicht untersucht □ |
| Sind weitere Komplikationen im Zusammenhang mit der initial verwendeten Blutstillungsmethode aufgetreten? | Ja □ Nein□ |
| Wenn ja, welche ? |  |
| Wie viele Tage (aufgerundet) hat der Patient auf Intensivstation verbracht nach initialer Blutstillung? |  |
| Wie viele Tage (aufgerundet) hat der Patient im Krankenhaus verbracht nach initialer Blutstillung? |  |
| Patient verstorben im 30-Tageintervall nach Intervention? | Ja □ Nein□ |
| Wenn verstorben, Todesursache ? |  |
| Konnten alle relevanten Studiendaten erhoben werden ? | Ja □ Nein□ |

**Bitte sende die Seite 5 umgehend nach Dokumentation per Fax/E-Mail an:**

**Fax +49-385-520-3726 oder Lucas.Thielemann**[**@helios-gesundheit.de**](mailto:Daniel.Schmitz@helios-gesundheit.de)
