# Supplementary material for: Bipolar haemostatic forceps versus standard therapy by haemoclip + / − epinephrine injection as initial endoscopic treatment in active non-variceal upper GI bleeding: study protocol for a prospective, randomized multicentre trial (BeBop-Trial)
Source: Trials. 2023 Jun 15;24:407. doi: 10.1186/s13063-023-07394-x (PMC10268387; doi:10.1186/s13063-023-07394-x)
Supplement: Supplementary file 2 — Additional file 2. Contract funder/coordinating study centre (German) from 31 December 2022. [file 13063_2023_7394_MOESM2_ESM.pdf]

DocuSign Envelope ID: E18E257F-04D0-42E4-B71A-260ED53A158C

Vertrag

über

die Unterstützung der klinischen Studie mit dem Titel

"Bipolare elektrische Blutstillungszange (HemoStat/Pentax) versus Standardtherapie (Clip +/- Unterspritzung mit Adrenalinlösung) bei der endoskopischen Erstbehandlung der aktiven nicht-varikösen Blutung im oberen Verdauungstrakt - BeBop"

---

zwischen der

Helios Kliniken GmbH
Friedrichstraße 136
10117 Berlin

vertreten durch die Geschäftsführung

- nachstehend „HELIOS" genannt -

und

Helios Kliniken Schwerin GmbH
Wismarsche Str. 393 – 397
19055 Schwerin

vertreten durch die Klinikgeschäftsführung

Studienleiter: Dr. med. Daniel Schmitz
Chefarzt der Klinik für Gastroenterologie und Infektiologie, Zentrum für Ultraschalldiagnostik und Interventionelle Endoskopie

- nachstehend „Studienleitung" genannt -

DocuSign Envelope ID: E18E257F-04D0-42E4-B71A-260ED53A158C

Präambel

Die Studienleitung beabsichtigt eine klinische Studie eigenverantwortlich durchzuführen. HELIOS unterstützt das Forschungsprojekt finanziell. Zu diesem Zweck wird folgende Vereinbarung getroffen.

§ 1 Vertragsgegenstand

(1) Gegenstand des Vertrages ist die klinische Studie mit dem Titel**:** " Bipolare elektrische Blutstillungszange (HemoStat/Pentax) versus Standardtherapie (Clip +/- Unterspritzung mit Adrenalinlösung) bei der endoskopischen Erstbehandlung der aktiven nicht-varikösen Blutung im oberen Verdauungstrakt - BeBop" (nachfolgend „Studie" genannt). Grundlage der Forschungsarbeiten ist der Prüfplan, der als **Anlage A** verbindlicher Vertragsbestandteil ist.

(2) HELIOS stellt zur Durchführung der Studie einen finanziellen Unterstützungsbeitrag in Höhe von **insgesamt EUR 22.530,00 brutto** zur Verfügung. Die aufgrund dieses Vertrages von HELIOS zur Verfügung gestellten Mittel sind Zuwendungen zur Erfüllung der Forschungsaufgaben des Studienleiters.

§ 2 Durchführung und Verantwortlichkeiten

(1) Die Studienleitung ist allein für die ordnungsgemäße Durchführung der Studie verantwortlich.

(2) Die Studie wird in Übereinstimmung mit den Vorgaben des Prüfplans (einschließlich sämtlicher Amendments) und nach Maßgabe aller anwendbaren Vorschriften durchgeführt, insbesondere nach dem Medizinproduktegesetzes (MPG), der EU-Datenschutzgrundverordnung (DSGVO), des Bundesdatenschutzgesetzes (BDSG) bzw. der anwendbaren Landesdatenschutzgesetze, der in der ICH GCP (Harmonised Tripartite Guideline for Good Clinical Practice) ausgewiesenen Empfehlungen sowie der Deklaration von Helsinki in den jeweils geltenden Fassungen.

(3) Die Studienleistung garantiert die Einhaltung aller anwendbaren gesetzlichen Erfordernisse, z.B. durch Einholung eines positiven Votums der zuständigen Ethikkommission (EK) und die erforderlichenfalls behördliche Meldung.

(4) Die Studienleitung sorgt bei jedem an der Studie teilnehmenden Patienten dafür, dass dieser umfassend über Wesen, Bedeutung und Tragweite der Studie aufgeklärt wird und holt die erforderliche schriftliche Einverständniserklärung ein.

(5) Die Zustimmung des endgültigen Prüfplans durch HELIOS ist eine Bedingung dafür, dass HELIOS diese Studie unterstützt. Über vorgesehene Änderungen an dem endgültigen Prüfplan wird HELIOS von der Studienleitung umgehend schriftlich informiert. Die fortlaufende Unterstützung der Forschungsarbeiten durch HELIOS setzt die Durchsicht und Zustimmung von HELIOS zu den Änderungen am Prüfplan voraus.

§ 3 Rechte an den Arbeitsergebnissen / Abschlussbericht / Erfindungen

(1) Die im Rahmen der Studie erarbeiteten Ergebnisse stehen im Eigentum der Studienleitung.

(2) HELIOS erhält ein Exemplar des wissenschaftlichen Ansprüchen genügenden Abschlussberichts. An den darin enthaltenen, nicht schutzrechtsfähigen Arbeitsergebnissen erhält HELIOS ein nicht ausschließliches, nicht übertragbares und nicht unterlizenzierbares Nutzungsrecht für interne Zwecke. Veröffentlichungen durch HELIOS, in denen Arbeitsergebnisse der Studie erwähnt/ verwendet werden sowie inhaltliche Änderungen oder auszugsweise Wiedergabe bedürfen der vorherigen schriftlichen Zustimmung durch die Studienleitung. Die Zustimmung wird nicht unbillig verweigert werden.

(3) Die Verwendung des Namens des Studienleiters außerhalb des Abschlussberichtes bzw. der üblichen Autorennennung (z.B. auf Werbematerialien) durch HELIOS bedarf der vorherigen schriftlichen Zustimmung der Studienleitung.

(4) Sollten anlässlich der Durchführung der Studie Erfindungen gemeldet werden, so stehen diese der Studienleistung zu. HELIOS kann an diesen Nutzungsrechte zu branchenüblichen Bedingungen erwerben. Die durch HELIOS im Rahmen dieser Studie geleistete finanzielle Unterstützung wird hierbei berücksichtigt. Einzelheiten werden zwischen den Parteien sodann in einer Zusatzvereinbarung festgelegt. Beide Parteien verpflichten sich, hierüber konstruktiv und zügig zu verhandeln.

§ 4 Veröffentlichungen

(1) Die Veröffentlichung der Studienergebnisse in einer anerkannten wissenschaftlichen Fachzeitschrift wird von der Studienleitung angestrebt. Grundsätzlich gilt, dass nur die Wissenschaftler als Autoren angegeben werden dürfen, welche einen eigenen Anteil geleistet haben. Insbesondere steht dem Studienleiter das Recht auf Ko-Autorenschaft zu. Im Übrigen orientieren sich die Parteien an den jeweils aktuellen Empfehlungen der Deutschen Forschungsgemeinschaft (DFG) zur „Sicherung guter wissenschaftlicher Praxis".

(2) Darüber hinaus ist HELIOS berechtigt, die im Abschlussbericht enthaltenen Studienergebnisse selbst zu veröffentlichen. Wenn die Studienleitung

DocuSign Envelope ID: E18E257F-04D0-42E4-B71A-260ED53A158C

gegenüber HELIOS ankündigen, dass sie beabsichtigen, die Studienergebnisse zu veröffentlichen, ist HELIOS zur Veröffentlichung der Studienergebnisse erst berechtigt, wenn die Studienleitung die Studienergebnisse vorveröffentlicht hat. Vorstehendes Vorveröffentlichungsrecht der Studienleitung ist beschränkt auf einen Zeitraum von einem (1) Jahr nach Übergabe des Abschlussberichts an HELIOS.

(3) Die Studienleitung verpflichtet sich, in den Veröffentlichungen auf die finanzielle Unterstützung durch HELIOS hinzuweisen. HELIOS erhält vor einer geplanten Veröffentlichung ein Manuskript zur Kommentierung. Änderungswünsche von HELIOS werden berücksichtigt, soweit sie den wissenschaftlichen Charakter oder die Neutralität der geplanten Veröffentlichung nicht beeinträchtigen. Änderungsvorschläge können nur berücksichtigt werden, wenn sie innerhalb von 30 Arbeitstagen nach Erhalt des Manuskriptes erhoben werden.

§ 5 Vertraulichkeit

(1) Die Parteien werden alle ihnen von der jeweils anderen Partei im Rahmen dieses Vertrages offen gelegten Informationen Dritten gegenüber vertraulich behandeln und nur zu dem in diesem Vertrag festgelegten Zweck verwenden. Die Parteien werden ihre Arbeitnehmer und sonstige Personen, die in die Erfüllung dieses Vertrages eingebunden sind, zu entsprechender Geheimhaltung verpflichten, soweit diese nicht bereits anderweitig vertraglich dazu verpflichtet sind.

(2) Eine darüberhinausgehende Verwendung und Weitergabe an Dritte bedarf der vorherigen schriftlichen Zustimmung der jeweils anderen Partei.

(3) Diese Verpflichtung bleibt auch über das Vertragsende hinaus wirksam.

§ 6 Gewährleistung und Haftung

(1) Die Studienleitung wird die Arbeiten sorgfältig und unter Einhaltung anerkannter wissenschaftlicher Standards durchführen. Die Parteien kennen das mit den Forschungsarbeiten verbundene Erfolgsrisiko. Die Studienleitung übernimmt aufgrund des Forschungscharakters der Arbeiten keinerlei Gewährleistung für das Erreichen eines bestimmten Arbeitsergebnisses oder dafür, dass das Arbeitsergebnis für einen bestimmten Zweck verwendet oder wirtschaftlich verwertet werden kann oder frei von Schutzrechten Dritter ist. Soweit entgegenstehende Schutzrechte bekannt werden, teilt die Studienleitung dies unverzüglich HELIOS mit.

(2) Die Haftung der Studienleitung und ihrer Mitarbeiter für Schäden von HELIOS, insbesondere für Schäden, die im Zusammenhang mit der Verwendung des Arbeitsergebnisses durch HELIOS entstehen, wird auf den

DocuSign Envelope ID: E18E257F-04D0-42E4-B71A-260ED53A158C

Unterstützungsbeitrag begrenzt. Die Haftungsbegrenzung gilt nicht für Schäden, die auf einer vorsätzlichen oder grob fahrlässigen Pflichtverletzung beruhen.

§ 7 Zahlungsweise, Verwendungsnachweis

(1) Der Unterstützungsbetrag gemäß § 1 (2) in Höhe von **insgesamt EUR 22.530,00** wird von HELIOS an die Studienleitung entsprechend dem Modus Rechnungsstellung (**Anlage B**), der Vertragsbestandteil ist, gegen Verwendungsnachweis ausbezahlt.

(2) Die Verwendung der Mittel durch die Studienleitung ist zweckgebunden und zur Deckung der von ihr übernommenen Ausgaben im Rahmen der Studie nach § 1 (1) vorgesehen.

(3) Die Verwendung des gesamten Unterstützungsbetrages ist HELIOS bis zum Abschluss der Studie von der Studienleitung nachzuweisen. Hierzu sind von der Studienleitung quartalsweise Zwischennachweise für den jeweils vorausgegangenen Zeitraum und am Ende ein Gesamtnachweis vorzulegen.

Der Verwendungsnachweis (**Anlage C**) besteht jeweils aus einem Sachbericht und einem zahlenmäßigen Nachweis:

- Der Sachbericht soll den Verlauf und die Ergebnisse der durchgeführten Forschungsarbeiten dokumentieren. Insbesondere ist hierbei herauszuarbeiten, inwieweit die Ziele und Teilziele erreicht werden konnten.
- Der zahlenmäßige Nachweis ist eine summarische Zusammenstellung der Einnahmen und Ausgaben entsprechend der Gliederung des Modus Rechnungsstellung (**Anlage B**). Auf Verlangen sind Originalbelege nachzureichen.

Im Übrigen orientieren sich die Nachweispflichten der Studienleitung an den Allgemeinen Nebenbestimmungen für Zuwendungen zur Projektförderung (ANBest-P) der öffentlichen Hand.

(4) Sollte die nach diesem Vertrag geschuldete Leistung der Studienleitung umsatzsteuerpflichtig sein, so ist die UW/H berechtigt, zusätzlich zu der in diesem Vertrag vereinbarten Vergütung die gesetzliche Umsatzsteuer zu fordern, wenn sie HELIOS eine Rechnung mit gesondertem Ausweis der Umsatzsteuer erteilt hat.

(5) Alle Zahlungen werden von HELIOS auf folgendes Konto der Studienleitung überwiesen:

Kontoinhaber: Helios Kliniken Schwerin GmbH

Bank: Commerzbank AG

IBAN: DE 10 1408 0000 0257 8050 00

BIC: DRESDEFF140

Verwendungszweck: HHI ID 2022-0035

§ 8 Vertragsdauer und Kündigung

(1) Dieser Vertrag wird mit dem Datum der letzten Unterschrift wirksam und bleibt ab dann für den geplanten Zeitraum der Studiendauer von 2 Jahren in Kraft. Er kann nach schriftlicher Absprache befristet verlängert werden. Den Zeitraum der Verlängerung und eine Kompensation möglicher zusätzlicher Kosten regeln die Parteien im gegenseitigen Einvernehmen mittels eines schriftlichen Amendments zum Vertrag. Kann bezüglich der Verlängerung keine einvernehmliche Einigung erzielt werden, so endet der Vertrag mit Ablauf der geplanten Gesamtstudiendauer von 2 Jahren.

(2) Der Vertrag kann von HELIOS mit einer angemessenen Frist nach vorheriger schriftlicher Mitteilung vorzeitig gekündigt werden, sofern:

- HELIOS den Prüfplanänderungen nicht zustimmt (vgl. § 2 Abs. 5).
- Eine unvorhergesehene wesentliche Kostenerhöhung eintritt, die die Parteien nicht einvernehmlich regeln.
- Der Fortgang der Forschungsarbeiten erheblich langsamer verläuft als im Prüfplan oder in der Antragstellung zur Unterstützung des Forschungsprojektes beschrieben, sodass die für die Studie geplanten Zeitvorgaben nicht erreicht werden.
- Die Forschungsziele der Studie nicht mehr länger wissenschaftlich relevant sind.

(3) Die außerordentliche Kündigung aus wichtigem Grunde bleibt beiden Parteien vorbehalten. Ein wichtiger Grund liegt insbesondere vor bei Vertragsbruch durch die jeweils andere Partei.

(4) Die Kündigung bedarf der Schriftform. HELIOS erhält einen Zwischennachweis, der die bis zur Vertragsbeendigung erzielten Arbeitsergebnisse beinhaltet.

(5) Erfolgt die Kündigung aus einem von der Studienleitung zu vertretenden Grund, der die Studienleitung oder HELIOS zur außerordentlichen Kündigung berechtigt, so erhält die Studienleitung eine anteilige finanzielle Unterstützung nur für die bis zum Beendigungszeitpunkt erbrachte Leistung (Zwischennachweis). Im Übrigen erstattet HELIOS über den Zeitpunkt der vorzeitigen Beendigung hinaus diejenigen Aufwendungen, die in Ansehung dieses Vertrages und in Erfüllung von Rechtspflichten noch anfallen, es sei denn, die Studienleitung unterlässt es pflichtwidrig, für die rechtzeitige Beendigung der rechtlichen Verpflichtungen Sorge zu tragen. Die über den Beendigungszeitpunkt hinaus zu erstattenden Aufwendungen dürfen die insgesamt für das Vorhaben veranschlagten Mittel nicht übersteigen.

§ 9 Sonstige Bestimmungen

(1) Der Vertrag unterliegt ausschließlich deutschem Recht. Das deutsche Kollisionsrecht findet keine Anwendung. Gerichtsstand ist Berlin.

(2) Abschluss, Änderungen und Ergänzungen dieses Vertrages sind entweder schriftlich (inkl. des E-Mail-Austausches eigenhändig unterzeichneter Unterschriftenseiten im pdf-Format) vorzunehmen oder durch den Austausch elektronischer Willenserklärungen im Rahmen von elektronischen Signaturverfahren durch renommierte Anbieter dieser Verfahren (z.B. DocuSign). Bei Nutzung elektronischer Signaturverfahren verpflichtet sich der Initiator/Account Owner des Signaturvorgangs allen Vertragsparteien den vom Anbieter erhältlichen offiziellen Unterzeichnungsbericht („Abschlussbericht" bei DocuSign) zur Verfügung zu stellen.

(3) Nebenabreden sind nicht getroffen.

§ 10 Salvatorische Klausel

Sollten einzelne Bestimmungen dieses Vertrages unwirksam sein, so wird die Gültigkeit der übrigen Bestimmungen dadurch nicht berührt. Anstelle der unwirksamen Bestimmung soll eine Regelung gelten, die dem am nächsten kommt, was die Vertragspartner gewollt haben oder gewollt hätten, wenn ihnen die Unwirksamkeit der Bestimmung bekannt gewesen wäre. Dasselbe gilt für etwaige Vertragslücken.

* * * * *

--- Unterschriften auf der nachfolgenden Seite ---

DocuSign Envelope ID: E18E257F-04D0-42E4-B71A-260ED53A158C

Für die Helios Kliniken GmbH

Berlin, den 14.09.2023 | 10:05:49 MESZ

DocuSigned by:
Robert Möller
18413EF21ECF427...
....
Robert Möller
Vorsitzender der Geschäftsführung (CEO) und
Geschäftsführer Medizin (CMO)

Berlin, den 14.09.2023 | 10:06:49 MESZ

DocuSigned by:
4C8BC3A2FD6844F...
....
Enrico Jensch
Geschäftsführer Operatives Geschäft (COO)

Für die Studienleitung:

Schwerin, den 17.09.2023 | 11:59:34 MESZ

DocuSigned by:
Daniel Dellmann
6C687427F9E04DF...
....
Daniel Dellmann, Klinikgeschäftsführer
Helios Kliniken Schwerin GmbH

Schwerin, den 17.09.2023 | 12:14:40 MESZ

DocuSigned by:
ECD1A0CEC679476...
....
Dr. Olaf Kannt, Klinikgeschäftsführer
Helios Kliniken Schwerin GmbH

Schwerin, den 18.09.2023 | 07:36:54 MESZ

DocuSigned by:
Daniel Schmitz
36B50C6028324E0...
....
Dr. med. Daniel Schmitz, Studienleiter
Chefarzt der Klinik für Gastroenterologie und Infektiologie,
Zentrum für Ultraschalldiagnostik und Interventionelle Endoskopie

DocuSign Envelope ID: E18E257F-04D0-42E4-B71A-260ED53A158C

Anlage A: Prüfplan, Version: 2.4 vom 19.12.2022

Anlage B: Modus Rechnungsstellung

Anlage C: Verwendungsnachweis
