# Supplementary material for: Bipolar haemostatic forceps versus standard therapy by haemoclip + / − epinephrine injection as initial endoscopic treatment in active non-variceal upper GI bleeding: study protocol for a prospective, randomized multicentre trial (BeBop-Trial)
Source: Trials. 2023 Jun 15;24:407. doi: 10.1186/s13063-023-07394-x (PMC10268387; doi:10.1186/s13063-023-07394-x)
Supplement: Supplementary file 3 — Additional file 3. Contract funder/coordinating study centre (translated in English) from 31 December 2022. [file 13063_2023_7394_MOESM3_ESM.pdf]

Contract

via

the support of the clinical study entitled

"Bipolar electric haemostatic forceps (HemoStat/Pentax) versus standard therapy (clip +/- subcutaneous injection with epinephrine solution) in the initial endoscopic treatment of active non-variceal haemorrhage in the upper digestive tract – BeBop-Trial"

between the

Helios Kliniken GmbH
Friedrichstrasse 136
10117 Berlin

represented by the management

- hereinafter referred to as "HELIOS" - and

and

Helios Kliniken Schwerin GmbH
Wismarsche Str. 393 - 397
19055 Schwerin

represented by the hospital management

Principal investigator: Dr. med. Daniel Schmitz
Head Physician of the Department of Gastroenterology and Infectiology, Centre for Ultrasound Diagnostics and Interventional Endoscopy

- hereinafter referred to as "Study Management".

Preamble

The study management intends to conduct a clinical study on its own responsibility. HELIOS supports the research project financially. For this purpose, the following agreement is made.

§ 1 Subject matter of the agreement

(1) The subject of the agreement is the clinical study entitled: " Bipolar electrical haemostasis forceps (HemoStat/Pentax) versus standard therapy (clip +/- subcutaneous injection with adrenaline solution) in the endoscopic initial treatment of active non-variceal haemorrhage in the upper digestive tract - BeBop" (hereinafter referred to as the "Study"). The basis of the research work is the study plan, which is a binding part of the contract (Appendix A).
(2) HELIOS shall provide a financial support contribution in the total gross amount of EUR 22,530.00 for the performance of the Study. The funds provided by HELIOS on the basis of this contract are grants for the fulfilment of the research tasks of the Principal Investigator.

§ 2 Conduct and responsibilities

(1) The Principal Investigator is solely responsible for the proper conduct of the study.
(2) The study will be conducted in accordance with the requirements of the protocol (including all amendments) and in compliance with all applicable regulations, in particular the German Medical Devices Act (MPG), the EU Data Protection Regulation (DSGVO), the German Federal Data Protection Act (BDSG) or the applicable state data protection laws, the recommendations stated in the ICH GCP (Harmonised Tripartite Guideline for Good Clinical Practice) as well as the Declaration of Helsinki in the respective applicable versions.
(3) The study performance guarantees compliance with all applicable legal requirements, e.g., by obtaining a positive vote of the competent Ethics Committee (EC) and the regulatory notification if necessary.
(4) The study management shall ensure that each patient participating in the study is fully informed about the nature, significance and scope of the study and obtains the required written informed consent.
(5) The approval of the final protocol by HELIOS is a condition for HELIOS to support this study. HELIOS will be promptly informed in writing by the study director of any proposed changes to the final protocol. HELIOS' continued support of the research is conditional upon HELIOS' review and approval of the changes to the protocol.

§ 3 Rights to the results of the work / final report / inventions

(1) The results produced within the framework of the study are the property of the study management.
(2) HELIOS shall receive a copy of the final report that meets scientific requirements. HELIOS shall receive a non-exclusive, non-transferable, and non-sublicensable right of use for internal purposes for the work results contained therein, which are not subject to intellectual property rights. Publications by HELIOS in which work results of the study are mentioned/used as well as changes in content or reproduction of

excerpts require the prior written consent of the study management. Consent will not be unreasonably withheld.
(3) The use of the name of the Principal Investigator outside the final report or the usual naming of authors (e.g., on advertising materials) by HELIOS requires the prior written consent of the study management.
(4) Should inventions be reported on the occasion of the performance of the study, the study performance shall be entitled to them. HELIOS may acquire rights of use to these inventions at conditions customary in the industry. The financial support provided by HELIOS within the scope of this study shall be considered. Details shall then be defined between the parties in a supplementary agreement. Both parties undertake to negotiate constructively and expeditiously.

§ 4 Publications

(1) The study management aims to publish the study results in a PubMed indexed scientific journal. In principle, only those scientists may be listed as authors who have made their own contribution. In particular, the Principal Investigator is entitled to co-authorship. In all other respects, the parties shall be guided by the respective current recommendations of the German Research Foundation (DFG) on "Safeguarding Good Scientific Practice".
(2) Furthermore, HELIOS shall be entitled to publish the study results contained in the final report itself. If the study management announce to HELIOS that they intend to publish the Study Results, HELIOS shall not be entitled to publish the Study Results until the Study Management has pre-published the Study Results. The aforementioned pre-publication right of the Study Management shall be limited to a period of one (1) year after delivery of the final report to HELIOS.
(3) The study management undertakes to refer to the financial support by HELIOS in the publications. HELIOS shall receive a manuscript for comment before a planned publication. Requests for changes by HELIOS will be considered as long as they do not affect the scientific character or the neutrality of the planned publication. Suggestions for changes can only be considered if they are raised within 30 working days after receipt of the manuscript.

§ 5 Confidentiality

(1) The parties shall treat all information disclosed to them by the respective other party within the scope of this contract as confidential vis-à-vis third parties and use it only for the purpose specified in this contract. The parties shall oblige their employees and other persons involved in the performance of this contract to observe the corresponding confidentiality, insofar as they are not already contractually obliged to do so elsewhere.
(2) Any further use and disclosure to third parties shall require the prior written consent of the respective other party.
(3) This obligation shall also remain effective beyond the end of the contract.

§ 6 Warranty and liability

(1) The study management will carry out the work carefully and in compliance with recognised scientific standards. The parties are aware of the risk of success associated with the research work. Due to the research character of the work, the study management does not assume any warranty for the achievement of a certain work result or that the work result can be used for a certain purpose or commercially exploited or that it is free of third-party property rights. Insofar as conflicting property rights become known, the study management shall inform HELIOS immediately.
(2) The liability of the study management and its employees for damages of HELIOS, in particular for damages arising in connection with the use of the work result by HELIOS, shall be limited to the support contribution. The limitation of liability shall not apply to damages resulting from an intentional or grossly negligent breach of duty.

§ 7 Mode of Payment, Proof of Use

(1) The Support Amount pursuant to § 1 (2) in the total amount of EUR 22,530.00 shall be paid by HELIOS to the Study Management in accordance with the mode of invoicing (Appendix B), which is part of the contract, against proof of use.
(2) The use of the funds by the Study Management is earmarked and intended to cover the expenses assumed by it within the scope of the Study pursuant to § 1 (1).
(3) The study management shall provide HELIOS with evidence of the use of the total amount of support by the end of the study. For this purpose, the study management shall submit quarterly interim proofs for the respective preceding period and a total proof at the end.
The proof of use (Appendix C) consists of a factual report and a numerical proof:
- The factual report shall document the course and results of the research work carried out. In particular, the extent to which the objectives and sub-objectives have been achieved must be elaborated.
- The numerical evidence shall be a summary of the income and expenditure according to the structure of the mode of accounting (Appendix B). Original receipts must be submitted upon request.
In all other respects, the study management's obligation to provide evidence is based on the General Auxiliary Conditions for Grants for Project Funding (ANBest-P) of the public sector.
(4) Should the performance of the study management owed under this contract be subject to VAT, UW/H shall be entitled to claim the statutory VAT in addition to the remuneration agreed in this contract if it has issued HELIOS with an invoice showing the VAT separately.
(5) All payments shall be transferred by HELIOS to the following account of the study management:

Account Holder: Helios Kliniken Schwerin GmbH Bank: Commerzbank AG
IBAN: DE 10 1408 0000 0257 8050 00
BIC: DRESDEFF140
Reason for payment: HHI ID 2022-0035

§ 8 Duration of contract and termination

(1) This contract becomes effective on the date of the last signature and remains in force from then on for the planned period of study of 2 years. It may be extended for a limited period after written agreement. The period of the extension and a compensation of possible additional costs shall be regulated by the parties in mutual agreement by means of a written amendment to the contract. If no mutual agreement can be reached regarding the extension, the contract ends with the expiry of the planned total study duration of 2 years.
(2) The contract may be terminated prematurely by HELIOS with a reasonable period of notice after prior written notification, provided that:
- HELIOS does not agree to the changes in the study plan (cf. § 2 para. 5).
- An unforeseen substantial increase in costs occurs which the parties do not settle by mutual agreement.
- The progress of the research work is considerably slower than described in the protocol or in the application for support of the research project, so that the time targets planned for the study are not achieved.
- The research objectives of the study are no longer scientifically relevant.
(3) Both parties reserve the right to extraordinary termination for good cause. Good cause shall be deemed to exist in particular in the event of breach of contract by the other party.
(4) The termination must be in writing. HELIOS shall receive an interim report containing the work results achieved until the termination of the contract.
(5) If the termination is due to a reason for which the study management is responsible and which entitles the study management or HELIOS to extraordinary termination, the study management shall receive pro rata financial support only for the work performed up to the date of termination (interim proof). Apart from that, HELIOS shall reimburse those expenses beyond the time of premature termination which are still incurred in view of this contract and in fulfilment of legal obligations, unless the study management fails in breach of duty to take care of the timely termination of the legal obligations. The expenses to be reimbursed beyond the termination date may not exceed the total funds budgeted for the project.

§ 9 Other provisions

(1) The contract shall be governed exclusively by German law. German conflict of laws shall not apply. The place of jurisdiction is Berlin.
(2) Conclusions, amendments and supplements to this contract shall be made either in writing (including the exchange of hand-signed signature pages in pdf format by e-mail) or by the exchange of electronic declarations of intent within the framework of electronic signature procedures by renowned providers of these procedures (e.g. DocuSign). When using electronic signature procedures, the initiator/account owner of the signature process undertakes to make the official signature report available from the provider ("final report" at DocuSign) available to all contracting parties.
(3) Ancillary agreements have not been made.

§ 10 Severability clause

Should certain conditions of this contract be invalid, this shall not affect the validity of the remaining conditions. In place of the invalid condition, a condition shall apply which comes as close as possible to what the contracting parties intended or would have intended if they had been aware of the invalidity of the condition. The same shall apply to any loopholes in the contract.

* * * * *

--- Signatures on the following page ---

For Helios Kliniken GmbH
Berlin, 14.09.2023 | 10:05:49 CEST

DocuSigned by:
Robert Möller
....
18413EF21ECF427...

Robert Möller
Chief Executive Officer (CEO) and Chief Medical Officer (CMO)

Berlin, 14.09.2023 | 10:06:49 CEST

DocuSigned by:
....
4C8BC3A2FD6844F...

Enrico Jensch
Chief Operating Officer (COO)

For the study management:
Schwerin, 17.09.2023 | 11:59:34 CEST

DocuSigned by:
Daniel Dellmann
....
6C687427F9E04DF...

Daniel Dellmann, Hospital Managing Director Helios Kliniken Schwerin GmbH

Schwerin, 17.09.2023 | _12:14:40 CEST

DocuSigned by:
....
ECD1A0CEC679476...

Dr. Olaf Kannt, Hospital Managing Director Helios Kliniken Schwerin GmbH
Schwerin, the_18.09.2023 | 07:36:54 CEST

DocuSigned by:
Daniel Schmitz
36B50C6028324E0...
....
Daniel Schmitz, MD, Principal Investigator
Head physician of the Clinic for Gastroenterology and Infectiology, Centre for Ultrasound Diagnostics and Interventional Endoscopy

Appendix A: Study plan, version: 2.4 of 19.12.2022
Appendix B: Invoicing mode
Appendix C: Where-used list
