# Supplementary material for: Bipolar haemostatic forceps versus standard therapy by haemoclip + / − epinephrine injection as initial endoscopic treatment in active non-variceal upper GI bleeding: study protocol for a prospective, randomized multicentre trial (BeBop-Trial)
Source: Trials. 2023 Jun 15;24:407. doi: 10.1186/s13063-023-07394-x (PMC10268387; doi:10.1186/s13063-023-07394-x)
Supplement: Supplementary file 4 — Additional file 4. Ethical vote (German) on 19 October 2022. [file 13063_2023_7394_MOESM4_ESM.pdf]

Universität Rostock Traditio et Innovatio

Universitätsmedizin Rostock
Eingang
19. Okt. 2022

Universitätsmedizin Rostock · PF 10 08 88 · 18055 Rostock

Helios Kliniken Schwerin GmbH
Klinik für Gastroenterologie und Infektiologie
Dr. med. Daniel Schmitz
Wismarsche Straße 393-397
19055 Schwerin

Ethikkommission an der Universitätsmedizin Rostock

Vorsitzender:
Univ.-Prof. Dr. med. Andreas Büttner
andreas.buettner@med.uni-rostock.de
Telefon: +49 381 494-9900

Geschäftsstellenleiterin:
Swaantje Kohlschein
kautz@med.uni-rostock.de
Telefon: +49 381 494-9904

Geschäftsstelle:
ethik@med.uni-rostock.de
Telefon: +49 381 494-9939
Fax: +49 381 494-9902
Homepage:
www.ethik.med.uni-rostock.de

19.10.2022

Stellungnahme der Ethikkommission

**Titel der Studie:**	**„Bipolare elektrische Blutstillungszange (HemoStat/Pentax) versus Standardtherapie (Clip+/- Unterspritzung mit Adrenalinlösung) bei der endoskopischen Erstbehandlung der aktiven nicht-varikösen Blutung im oberen Verdauungstrakt – BeBop-Studie“**
**Studienleiter vor Ort:**	**Dr. med. Daniel Schmitz<br>Helios Kliniken Schwerin GmbH<br>Klinik für Gastroenterologie und Infektiologie<br>Wismarsche Straße 393-397<br>19055 Schwerin**

Registriernummer: (Bei Schriftwechsel bitte stets angeben) **A 2022-0166**
Eingang Ethikkommission: 12.10.2022

Sehr geehrter Herr Dr. med. Daniel Schmitz,

der Vorsitzende der Ethikkommission an der Universitätsmedizin Rostock hat die von Ihnen eingereichten Unterlagen im Auftrag der Kommission geprüft. Die Unterlagen liegen der Kommission vollständig vor.

Es bestehen aus **berufsrechtlicher und ethischer Sicht keine Bedenken** gegen die Durchführung des o.g. Forschungsprojekts.

Wir weisen Sie darauf hin, dass die ärztliche und juristische Verantwortung des Leiters des Projektes und der teilnehmenden Ärzte entsprechend der Beratungsfunktion der Ethikkommission von dieser Stellungnahme unberührt bleibt.

Folgende Dokumente lagen uns zur Bewertung vor:

1. **Anschreiben vom 07.10.2022**
2. **Checkliste Ethik Rostock 2022-10-5**
3. **CRF BeBop Vs 2.1 vom 17.9.2022.**
4. **PatInfo BeBop Vs 2.1 vom 17.9.2022.**
5. **Prüfplan BeBop Vs. 2.1 vom 6.10.2022**

Allgemeine Hinweise:

1. Die ethische und rechtliche Verantwortung für die Durchführung dieser klinischen Prüfung verbleibt beim Sponsor, bei der Leiterin/dem Leiter der klinischen Prüfung und bei den Prüferinnen/Prüfern.
2. Zusammensetzung und Arbeitsweise der Ethik-Kommission entsprechen nationalen Gesetzen, Vorschriften und der ICH-GCP-Leitlinie in der jeweils gültigen Fassung.
3. Datenschutzrechtliche Aspekte von Forschungsvorhaben werden durch die Ethikkommission grundsätzlich nur kursorisch geprüft. Dieses Votum / diese Bewertung ersetzt mithin nicht die Konsultation des zuständigen Datenschutzbeauftragten.

Mit freundlichen Grüßen

Univ.-Prof. Dr. med. A. Büttner
Vorsitzender der Ethikkommission

Thomas Penno
Geschäftsstelle

Anlage:
Mitgliederliste der Ethikkommission

Mitglieder der Ethikkommission an der Universitätsmedizin Rostock

Vorsitz:

Herr Prof. Dr. med. Andreas Büttner
Facharzt für Rechtsmedizin

Mitglieder:

Herr Prof. Dr. Günther Kundt
Biometriker

Herr Dr. jur. Markus Glöckner
Jurist

Frau Katrin Jeremias
Krankenhausseelsorgerin

Herr PD Dr. med. Felix Meinel
Facharzt für Radiologie

Frau Dr. med. Silke Müller
Fachärztin für Klinische Pharmakologie

Herr Prof. Dr. med. Carl-Friedrich Classen
Facharzt für Kinder- und Jugendmedizin

Herr Dr. med. Michael Bolz
Facharzt für Frauenheilkunde / Geburtshilfe

Herr Prof. Dr. med. Gerhard Stuhldreier
Facharzt für Chirurgie
Facharzt für Kinderchirurgie

Frau Dr. med. Brigitte Kragl
Fachärztin für Innere Medizin

Prof. Dr. med. Carsten Spitzer
Facharzt für Psychosomatische Medizin und Psychotherapie
Facharzt für Psychiatrie und Psychotherapie

Prof. Dr. Hermann Lang
Zahnmediziner
Facharzt für Konservierende Zahnheilkunde
