# Supplementary material for: Bipolar haemostatic forceps versus standard therapy by haemoclip + / − epinephrine injection as initial endoscopic treatment in active non-variceal upper GI bleeding: study protocol for a prospective, randomized multicentre trial (BeBop-Trial)
Source: Trials. 2023 Jun 15;24:407. doi: 10.1186/s13063-023-07394-x (PMC10268387; doi:10.1186/s13063-023-07394-x)
Supplement: Supplementary file 5 — Additional file 5. Ethical vote (translated in English) on 19 October 2022. [file 13063_2023_7394_MOESM5_ESM.docx]

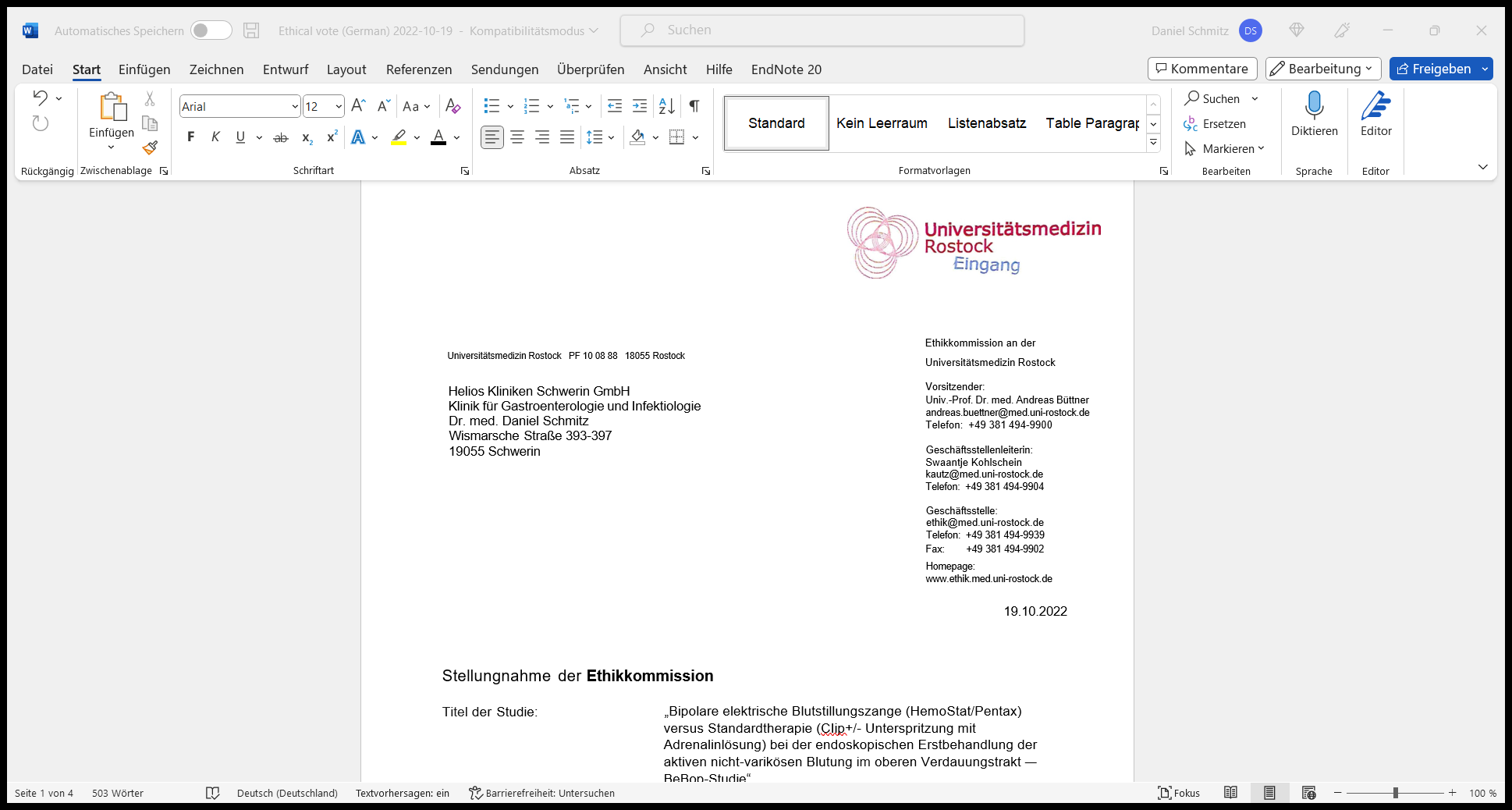


**Statement of the Ethical Committee**

Study title:

"Bipolar haemostatic forceps (HemoStat/Pentax) versus standard therapy (CIip+/- injection with epinephrine solution) in the initial endoscopic treatment of active non-variceal haemorrhage in the upper digestive tract - BeBop study".

Principal Investigator:

Daniel Schmitz, MD

Helios Kliniken Schwerin GmbH

Department of Gastroenterology and Infectiology, Wismarsche Straße 393-397

19055 Schwerin

Registration number: (Please always quote in all correspondence) A 2022-0166 Received by Ethical Committee on 12 October 2022Dear Daniel Schmitz, MD,

The chairman of the Ethics Committee at Rostock University Medical School has examined the documents you submitted on behalf of the committee. The documents are available to the commission in their entirety.

From a professional and ethical point of view, there are no objections to the implementation of the above-mentioned research project.

We would like to point out that the medical and legal responsibility of the head of the project and the participating doctors remains unaffected by this opinion, in accordance with the advisory function of the ethics committee.

The following documents were available for evaluation:

1. Cover letter dated 07.10.2022

2. Checklist ethic committee Rostock 2022-10-5

3. CRF BeBop Vs 2.1 dated 17.9.2022.

4. Patient information BeBop Vs 2.1 of 17.9.2022.

5. Study protocol BeBop Vs 2.1 dated 6.10.2022

General information:

1. the ethical and legal responsibility for the conduct of this trial remains with the sponsor, the principal investigator, and the investigators.

2. the composition and working methods of the ethics committee are in accordance with national laws, regulations and the ICH-GCP guideline as amended from time to time.

3. data protection aspects of research projects are only examined by the ethics committee in a cursory manner. This vote/assessment therefore does not replace consultation with the competent data protection officer.

With best regards,


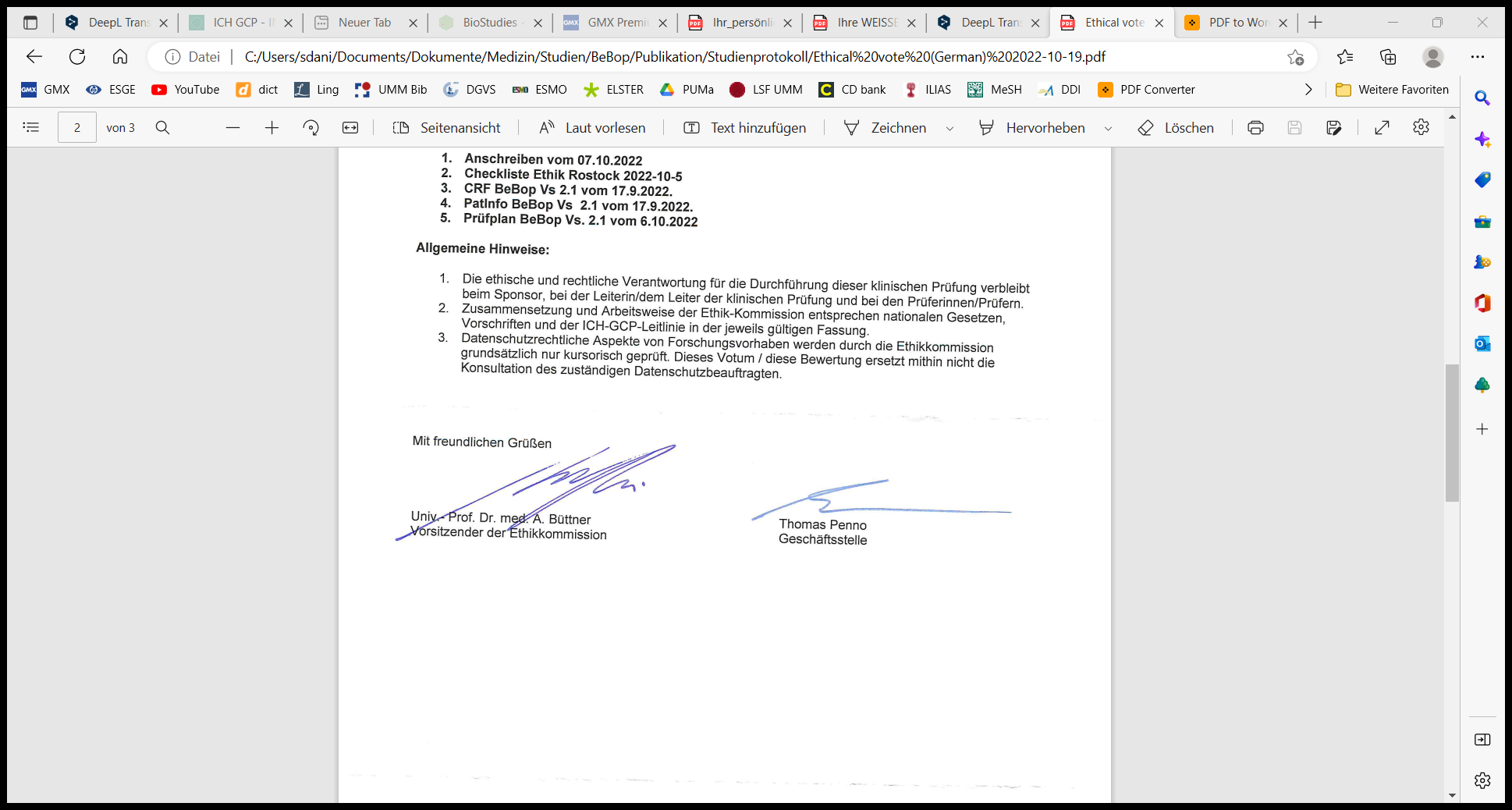


Members of the Ethics Committee:

Prof. Dr. med. Andreas Büttner (Chairman)

Specialist in Forensic Medicine

Prof. Dr. Günther Kundt

Biometrician

Dr. jur. Markus Glöckner

Lawyer

Mrs Katrin Jeremias

Hospital chaplain

Prof. Dr. med. Felix Meinel

Specialist in Radiology

Ms Dr. med. Silke Müller

Specialist in Clinical Pharmacology

Prof. Dr. med. Carl-Friedrich Classen

Specialist in Paediatrics and Adolescent Medicine

Mr Dr. med. Michael Bolz

Specialist in gynaecology / obstetrics

Prof. Dr. med. Gerhard Stuhldreier Specialist in Surgery

Specialist in paediatric surgery

Brigitte Kragl, MD

Specialist in Internal Medicine

Prof. Dr. med. Carsten Spitzer

Specialist in Psychosomatic Medicine and Psychotherapy Specialist in Psychiatry and Psychotherapy

Prof. Dr. Hermann Lang

Dentist

Specialist in conservative dentistry
