# Supplementary material for: Bipolar haemostatic forceps versus standard therapy by haemoclip + / − epinephrine injection as initial endoscopic treatment in active non-variceal upper GI bleeding: study protocol for a prospective, randomized multicentre trial (BeBop-Trial)
Source: Trials. 2023 Jun 15;24:407. doi: 10.1186/s13063-023-07394-x (PMC10268387; doi:10.1186/s13063-023-07394-x)
Supplement: Supplementary file 7 — Additional file 7. Ethical vote amendment (translated in English) on 16 January 2023. [file 13063_2023_7394_MOESM7_ESM.docx]

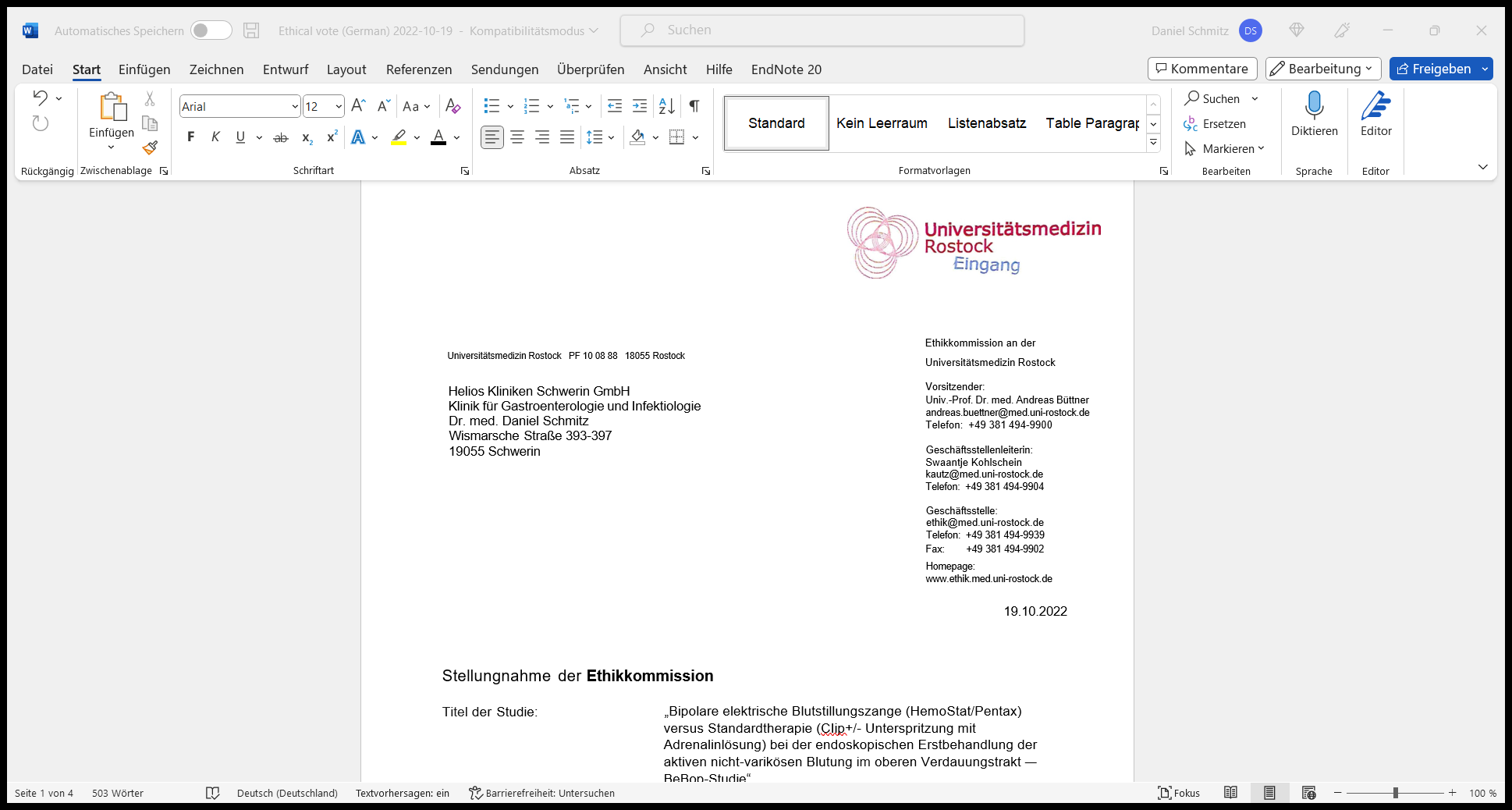


16.01.2023

**Statement of the Ethical Committee**

Study title:

"Bipolar haemostatic forceps (HemoStat/Pentax) versus standard therapy (CIip+/- injection with epinephrine solution) in the initial endoscopic treatment of active non-variceal haemorrhage in the upper digestive tract - BeBop study".

Principal Investigator:

Daniel Schmitz, MD

Helios Kliniken Schwerin GmbH

Department of Gastroenterology and Infectiology, Wismarsche Straße 393-397

19055 Schwerin

Registration number: (Please always quote in all correspondence) A 2022-0166.

Entry Ethics Committee: 09.01.2023 (digital)

Dear Daniel Schmitz, MD,

The Ethics Committee of the University Medical Centre Rostock has received further documents regarding the above-mentioned study in a letter dated 09.01.2023. The committee acknowledges reception of the following documents and acknowledges them:

1. CRF BeBop Vs 2.2 dated 03.12.2022 with markings.

2. CRF BeBop Vs 2.2 dated 03.12.2022_finaI.

3. Patient information BeBop Vs 2.3 of 12.12.2022 with markings.

4. Patient information BeBop Vs 2.3 of 12.12.2022_finaI.

5. Study protocol BeBop Vs. 2.4 of 30.12.2022 with markings

6. protocol BeBop Vs. 2.4 of 30.12.2022-_final

From a professional and ethical point of view, there are no objections to the continuation of the above-mentioned research project.

We would like to point out that the medical and legal responsibility of the project leader and the participating physicians in terms of the advisory function of the ethics committee remains unaffected by this statement.

General information:

1. the ethical and legal responsibility for the conduct of this clinical trial remains with the sponsor, the clinical trial director, and the investigators.

2. the composition and functioning of the Ethics Committee shall be in accordance with national laws, regulations and the ICH GCP guideline as amended from time to time. 3.

3. data protection aspects of research projects are only examined cursorily by the ethics committee. This vote/assessment therefore does not replace consultation with the responsible data protection officer.

With best regards,


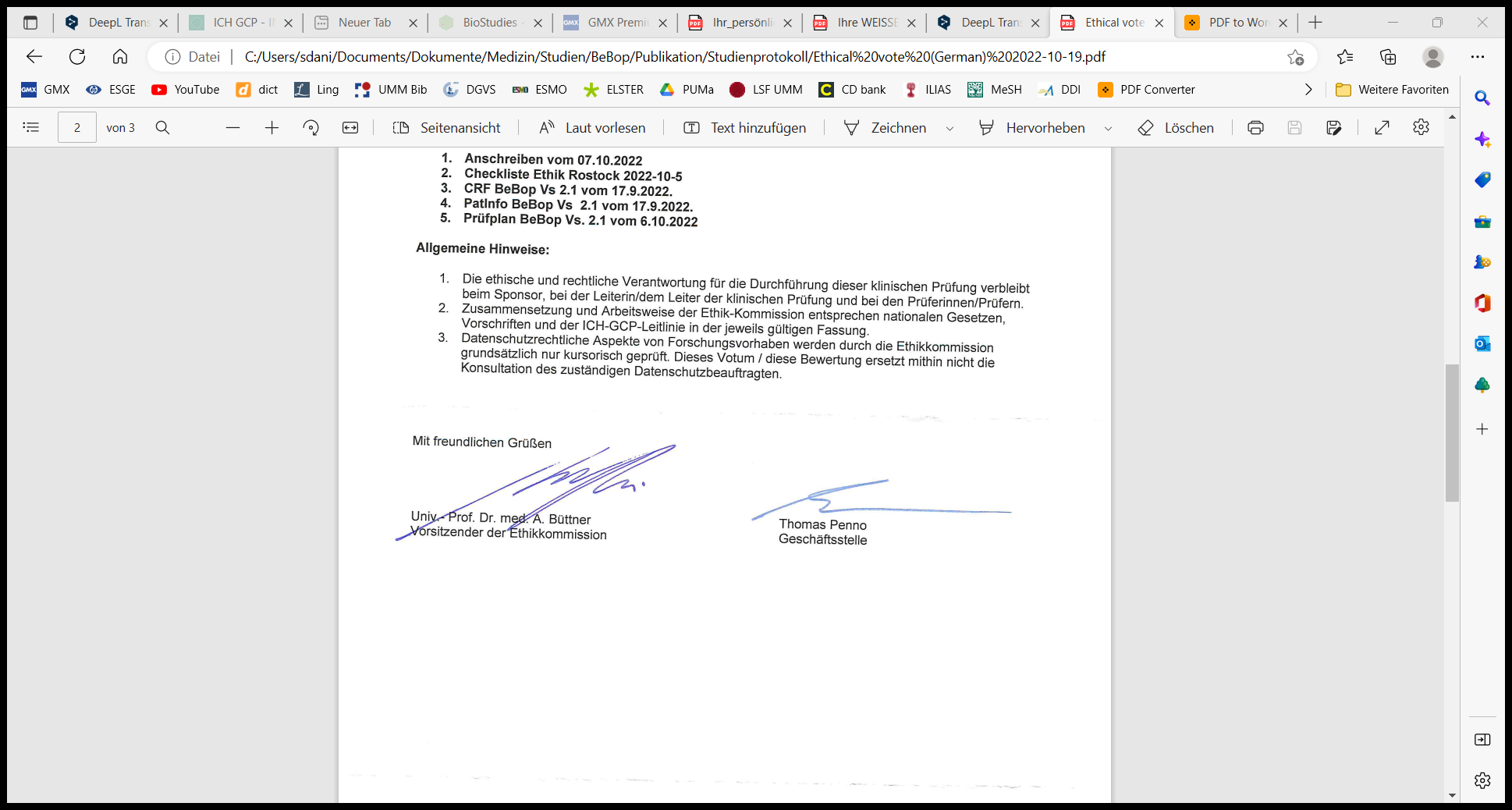


Members of the Ethics Committee:

Prof. Dr. med. Andreas Büttner (Chairman)

Specialist in Forensic Medicine

Prof. Dr. Günther Kundt

Biometrician

Dr. jur. Markus Glöckner

Lawyer

Mrs Katrin Jeremias

Hospital chaplain

Prof. Dr. med. Felix Meinel

Specialist in Radiology

Ms Dr. med. Silke Müller

Specialist in Clinical Pharmacology

Prof. Dr. med. Carl-Friedrich Classen

Specialist in Paediatrics and Adolescent Medicine

Mr Dr. med. Michael Bolz

Specialist in gynaecology / obstetrics

Prof. Dr. med. Gerhard Stuhldreier Specialist in Surgery

Specialist in paediatric surgery

Brigitte Kragl, MD

Specialist in Internal Medicine

Prof. Dr. med. Carsten Spitzer

Specialist in Psychosomatic Medicine and Psychotherapy Specialist in Psychiatry and Psychotherapy

Prof. Dr. Hermann Lang

Dentist

Specialist in conservative dentistry
